# Expression patterns and promoter analyses of aluminum-responsive *NAC* genes suggest a possible growth regulation of rice mediated by aluminum, hormones and NAC transcription factors

**DOI:** 10.1371/journal.pone.0186084

**Published:** 2017-10-12

**Authors:** Hugo Fernando Escobar-Sepúlveda, Libia Iris Trejo-Téllez, Soledad García-Morales, Fernando Carlos Gómez-Merino

**Affiliations:** 1 Department of Biotechnology, Colegio de Postgraduados Campus Córdoba, Manuel León, Amatlán de los Reyes, Veracruz, Mexico; 2 Department of Soil Science, Laboratory of Plant Nutrition, Colegio de Postgraduados Campus Montecillo, Montecillo, Texcoco, State of Mexico, Mexico; 3 Department of Plant Biotechnology, CONACYT-Center for Research and Assistance in Technology and Design of the State of Jalisco, Zapopan, Jalisco, Mexico; National Taiwan University, TAIWAN

## Abstract

In acid soils, the solubilized form of aluminum, Al^+3^, decreases root growth and affects the development of most crops. However, like other toxic elements, Al can have hormetic effects on plant metabolism. Rice (*Oryza sativa*) is one of the most tolerant species to Al toxicity, and when this element is supplied at low doses, growth stimulation has been observed, which could be due to combined mechanisms that are partly triggered by NAC transcription factors. This protein family can regulate vital processes in plants, including growth, development, and response to environmental stimuli, whether biotic or abiotic. Under our experimental conditions, 200 μM Al stimulated root growth and the formation of tillers; it also caused differential expression of a set of *NAC* genes. The promoter regions of the genes regulated by Al were analyzed and the *cis*-acting elements that are potentially involved in the responses to different stimuli, including environmental stress, were identified. Through the Genevestigator platform, data on the expression of *NAC* genes were obtained by experimental condition, tissue, and vegetative stage. This is the first study on *NAC* genes where *in vivo* and *in silico* data are complementarily analyzed, relating the hormetic effect of Al on plant growth and gene expression with a possible interaction in the response to phytohormones in rice. These findings could help to elucidate the possible convergence between the signaling pathways mediated by phytohormones and the role of the NAC transcription factors in the regulation of growth mediated by low Al doses.

## Introduction

Crop productivity and sustainability are key elements in food security. Nevertheless, crops are negatively affected by different types of stress, including aluminum (Al) toxicity. Al is an important constituent of soils; it is the third most abundant element in the Earth’s crust, after oxygen (O) and silicon (Si), making up 7% of the mass [1]. Under acid soil conditions (pH < 5.0), Al adopts a trivalent form, Al^+3^, which is toxic to plants, especially when found in high concentrations [2]. In many studies on the mechanisms of Al toxicity, it is proven that this element acts in several cell sites in the roots [3–6]. Overexposure to Al mainly produces a decrease in root growth, which implies a concomitant reduction in water and nutrient absorption from the soil [7]. Approximately 30 to 50% of all arable land exhibits acid conditions, so Al toxicity is an abiotic stress factor that hinders agricultural productivity [8,9].

Cereals have different levels of Al tolerance [2,10,11]. Among them, rice (*Oryza sativa*) is the species that has developed the most efficient mechanisms to tolerate toxic levels of this metal [10]; and between genotypes of this species, the *japonica* subspecies is significantly more tolerant than the *indica* subspecies [12]. Among these tolerance mechanisms, the regulation of gene expression in response to Al is crucial to achieve survival [13] and the transcription factors play a key role in this regulation. The *ASR* genes (*ASR1* and *ASR5*) are one of the pivotal components in the Al toxicity response machinery, as they codify transcription factors in rice, but they are absent in Arabidopsis [14]. When rice is exposed to toxic levels of Al, the ASR transcription factors act concertedly and complementarily, recognizing the *cis*-acting elements in the promoters of the *STAR1* gene to potentiate the Al response expression of a set of target genes [15–17]. Another family of transcription factors that responds to Al is NAC [1,18], which are specific to plants [19–21]. The acronym, NAC, derives from three genes that codify the NAC domain: *NAM* (for *no apical meristem*), *ATAF1/2* (for *Arabidopsis thaliana Activation Factor 1/2*), and *CUC2* (for *cup-shaped cotyledon 2*). They have multiple cell functions, including growth and development regulation, as well as responses to environmental stimuli like heat, cold, salinity, drought, and Al [1,18,22–26].

Unlike Al toxicity, research works on the beneficial effects of Al on plants are scarce. In rice, the first report indicated that Al stimulates growth [27]; later, in an analysis by the genome-wide association (GWA) on 383 different rice accessions in response to Al, 16 cultivars were reported to show an increase in root growth [12]. Subsequently, Al was reported to have increased the concentration of chlorophylls (*a* and *b*) and carotenoids [28]. Recently, it was observed that 200 μM Al in 4 cultivars (Cotaxtla, Tres Ríos, Huimanguillo, and Temporalero) stimulated growth, increased the concentrations of chlorophylls and total soluble sugars in the plantlet, and augmented P and K concentrations in the root [1]. Beneficial effects of Al have also been reported in other crops. In maize (*Zea mays*), for example, leaf growth stimulation was observed [29], and in soybean (*Glycine max*), Al increased seedling shoot and root growth, as well as antioxidant activity [30]. Importantly, the NAC transcription factors may mediate root growth and development triggered by Al. The first study suggesting a possible involvement of *NAC* genes in Al responses reported that the *OsNAC5* (*Os11g08210*) gene is responsive to this metal in roots of maize plants exposed to Al for 24 h [18]. Moreover, differential expression in a group of 25 *NAC* genes in the roots of four different rice cultivars in response to Al stimuli was also observed [1].

Although gene expression can be regulated at the transcriptional, post-transcriptional, and post-translational levels, transcriptional regulation is more responsible in the activation and repression of the transcription of one or more genes, and is controlled through gene promoters and their corresponding *cis*-acting elements [31]. Promoters are DNA sequences located upstream of the gene codifying regions, and they contain several *cis*-acting elements, which are specific binding sites for proteins involved in the initiation and regulation of transcription. The identification of the *cis*-acting elements in the promoters allows them to be used as an essential tool for the detection of gene expression patterns in response to a determined factor [32]. In rice, 20 *NAC* stress-inducible genes were found, in whose promoter regions *cis*-acting elements were identified in response to abscisic acid (ABRE) [33], dehydration (DRE) [34], and low temperatures (LTR) [35]. To date, no *cis*-acting elements have been reported in the promoter regions of Al-regulated *NAC* genes. Al generates complex metabolic responses [36,37], where phytohormones like auxin [38], ethylene [38,39], and jasmonic acid [40] intervene. Together, these signaling molecules may mediate stimulation or inhibition of root growth and development, depending on whether Al is found in beneficial or toxic concentrations, respectively. Therefore, in the present study we evaluated the effect of Al on growth and the expression of 57 *NAC* genes in four rice cultivars. Moreover, all relevant *cis*-acting elements and putative motifs responsive to phytohormones were determined to prove the role of *NAC* genes in response to Al, through the analysis of promoters. We also analyzed data of expression profiles of *NAC* genes in the presence of different phytohormones through the Genevestigator platform (https://genevestigator.com/gv/index.jsp) [41].

This study is relevant because it shows, for the first time, that the beneficial effect of Al on the growth of rice plants is mediated by *NAC* transcription factors that respond to phytohormones. The promoter regions of the Al-induced *NAC* genes were shown to contain *cis*-acting elements that respond to auxins, cytokinins, gibberellins, abscisic acid, and ethylene. The information derived from this study may be useful for the design of strategies for the use of Al as a biostimulant of growth in rice and other plants, which is mediated by phytohormones.

## Materials and methods

### Plant material and plant growth conditions

Four rice (*Oryza sativa* L. ssp. *indica*) cultivars were used: Cotaxtla, Tres Ríos, Huimanguillo, and Temporalero, which were obtained from the Germplasm Bank of the National Institute of Forest, Agricultural, and Livestock Research (Instituto Nacional de Investigaciones Forestales, Agrícolas y Pecuarias—INIFAP), located in the Zacatepec Experimental Station, in the state of Morelos, Mexico (18°39’ NL, 99°12’ WL, 910 masl). The four cultivars evaluated were selected based on their contrasting performance when exposed to different environmental cues, including Al [1,42]. These cultivars were produced for establishment in the tropical soils of Mexico, which display different degrees of acidity. The seeds of these cultivars were disinfected and germinated according to García-Morales et al. (2014) [43]. Eleven days after germination, plants were transplanted in containers with 12 L Yoshida nutrient solution which contained 1.43 mM NH_4_NO_3_, 1.00 mM CaCl_2_ 2H_2_O, 1.64 mM MgSO_4_ 7H_2_O, 1.30 mM K_2_SO_4_, 0.32 mM NaH_2_PO_4_ 2H_2_O, 1.00 mM Fe-EDTA, 7.99 μM MnCl_2_ 4H_2_O, 0.15 μM ZnSO_4_ 7H_2_O, 0.15 μM CuSO_4_ 5H_2_O, 0.075 μM (NH_4_)_6_Mo_7_O_24_ 4H_2_O, and 1.39 μM H_3_BO_3_. Thirteen days after transplantation, the nutrient solution was completely replaced and rice plants were placed in the control treatment (without Al) or the treatment with Al (200 μM AlCl_3_, pH 4.2) for 20 days. The hydroponic solution was renewed every 4 days. This experimental stage was carried out under greenhouse conditions with a 12 hour photoperiod at 30/20°C (day/night), 40/80% relative humidity (day/night), and 300 μmol m^-2^ s^-1^ light intensity.

### Plant growth

After 20 days of exposure to either 0 or 200 μM Al, plants were harvested and measured. Plant height was determined by measuring from the base of the shoot to the tip of the flag leaf. Root growth was assessed by measuring from the base of the shoot to the tip of the longest root. Relative growth was estimated by dividing the shoot and root growth values with Al by the growth in the control plants (without Al) x 100%. Also, the number of tillers and root volume were determined.

### Quantitative RT-PCR analyses

For gene expression analyses, rice seedlings were grown as described above. Twenty-four hours after exposure to either control or Al treatment, plants were collected and separated into shoot and root; each replicate was represented by the shoots and roots of three individual plants. Three independent biological replicates were immediately frozen in liquid nitrogen and stored at -80 ºC. RNA extraction, cDNA synthesis, and qRT-PCR were carried out as described by García-Morales et al. (2014) [43] and Moreno-Alvarado et al. (2017) [1]. The primers for the NAC transcription factors were those previously used by García-Morales et al. (2014) [43] and selected from those reported by Caldana et al. (2007) [44]. In this study we also included the genes *OsNAC6* [45], *OsNAC5* [46], and *OsNAC10* [47]. Furthermore, three control genes were also taken into consideration for the tests, which are known to respond to Al: *STAR1*, *ASR5* [15,17,48], and *OsNAC5* [18]. The reference genes evaluated were *actin*, *actin 1*, *β-tubulin*, and *elongation factor 1α*. The expression stability values (*M*) of all reference genes were estimated in accordance with Vandesompele et al. (2002) [49]. *Actin* was selected as the reference gene, which displayed the lowest *M* value. All the reactions were done with three technical replicates. The relative expression of the genes was determined using the 2^-ΔΔC^_T_ method [50]. The genes were considered as induced or repressed with an absolute value of ≥ 2.0. The primer pairs used in this study are listed in **S1 Table**.

### Multiple alignment of protein sequences

For this analysis we used the protein sequences of the NAC transcription factors of rice ssp. *japonica* tested in our *in vivo* study (Table 1). Given that the expression of *NAC* genes was evaluated in cultivars of *Oryza sativa* ssp. *indica* under our experimental conditions (i.e. in the cultivars Cotaxtla, Tres Ríos, Huimanguillo and Temporalero), we considered the sequences of the differentially regulated genes, which were obtained from the following databases: PlantTFDB v4.0 (http://planttfdb.cbi.pku.edu.cn/) [51] and PlnTFDB v3.0 (http://plntfdb.bio.uni-potsdam.de/v3.0/) [52]. The multiple alignments for the comparison of protein sequences between NAC of the *japonica* and *indica* subspecies were done using the blastp v2.6.0 software (https://blast.ncbi.nlm.nih.gov/Blast.cgi) [53]. For this analysis, we used all the default parameters set by the program.

**Table 1:** Groups of *NAC* genes classified according to their expression pattern in roots, shoots, or both tissues in 200 μM Al treated rice plants^a^.

Specific expression in roots	Specific expression in shoots	Expression in both roots and shoots
*Os03g60080*	*Os03g56580*	*Os02g56600*
*Os01g15640*	*Os06g46270*	*Os03g21060*
*Os09g32040*	*Os03g03540*	*ma1*
*Os12g43530*	*Os03g02800*	*Os10g42130*
*Os06g51070*	*Os03g01870*	*Os01g66490*
*Os11g31330*	*Os06g01480*	*Os07g04560*
*Os04g35660*	*Os02g34970*	*Os09g33490*
*Os03g59730*	*Os01g59640*	*Os07g13920*
	*Os11g04960*	*Os10g21560*
	*Os06g15690*	*Os04g40130*
	*Os12g07790*	*Os08g10080*
	*Os10g27360*	*Os12g29330*
	*Os03g42630*	*Os04g38720*
	*Os02g36880*	*Os01g66120*
	*Os11g03300*	*Os01g48446*

^a^The genes were obtained from *Oryza sativa* ssp. *japonica* and subsequently evaluated in *Oryza sativa* ssp. *indica*. The IDs are shown. ma = missing annotation.

### Acquisition of the rice *NAC* gene promoters

We considered the *Osxxgxxxxx*.*x* identifiers of *NAC* genes of *Oryza sativa* ssp. *japonica* tested in our *in vivo* study (Table 1) where they were necessary and sufficient to obtain their respective promoters. These nucleotide sequences were downloaded from the TIGR v6.0 platform (ftp://ftp.plantbiology.msu.edu/pub/data/Eukaryotic_Projects/o_sativa/annotation_dbs/pseudomolecules/version_6.0/all.dir/), considering 1000 base pairs (bp) upstream of the start codon. All promoter sequences analyzed are listed in **S1 File.**

### Promoter analysis

The *cis*-acting elements in each promoter were revealed through the PlantCARE database (http://bioinformatics.psb.ugent.be/webtools/plantcare/html/) [54], while the putative motifs were determined through the MEME v4.11.2 software (http://meme-suite.org/tools/dreme) [55]. In the case of the latter software, the motif with E-value < 0.01 and displaying lengths varying from 6 to 10 bp was chosen for each group of sequences. According to the differential expression in roots, leaves, and both tissues (result of the *in vivo* experiments done in this research on *NAC* genes regulated by Al; Table 1), the sequences were divided into three groups. Since we expected the sequences of the motifs to repeat more than once, the frequency of their distribution was not considered as a parameter in our analysis. For a better visualization of the distribution of the motifs with respect to the bp where they are located in the sequences, they were aligned through the MAST v4.11.2 software (http://meme-suite.org/tools/mast) [56]). Moreover, the Tomtom v4.11.2 software (http://meme-suite.org/tools/tomtom) [57] was used with the JASPAR DNA CORE (2016) plant motifs database to identify the function of each motif, using the Pearson correlation coefficient with a significance threshold to the E-value lower than 10.

### Analysis of expression profiles

The expression profiles data were obtained through the Genevestigator platform (https://genevestigator.com/gv/index.jsp) [41], where the experiments under the “Hormone” section were selected. The search for experiments was done using the keyword NAC. For this analysis, we considered all the *NAC* genes tested *in vivo* in this work (Table 1), plus the genes *OsNAC5* (*Os11g08210*), *ASR5* (*Os11g06720*), and *STAR1* (*Os06g48060*), which served as positive controls.

### Statistical analysis

For the growth data, an analysis of variance was done using the SAS [58] statistical software, and mean comparison with the Tukey test, with *P* ≤ 0.05. For gene expression, the Fisher LSD (*P* ≤ 0.05) test was used to obtain the separation of means.

## Results and discussion

### Aluminum stimulates plant growth in rice

**Fig 1:**
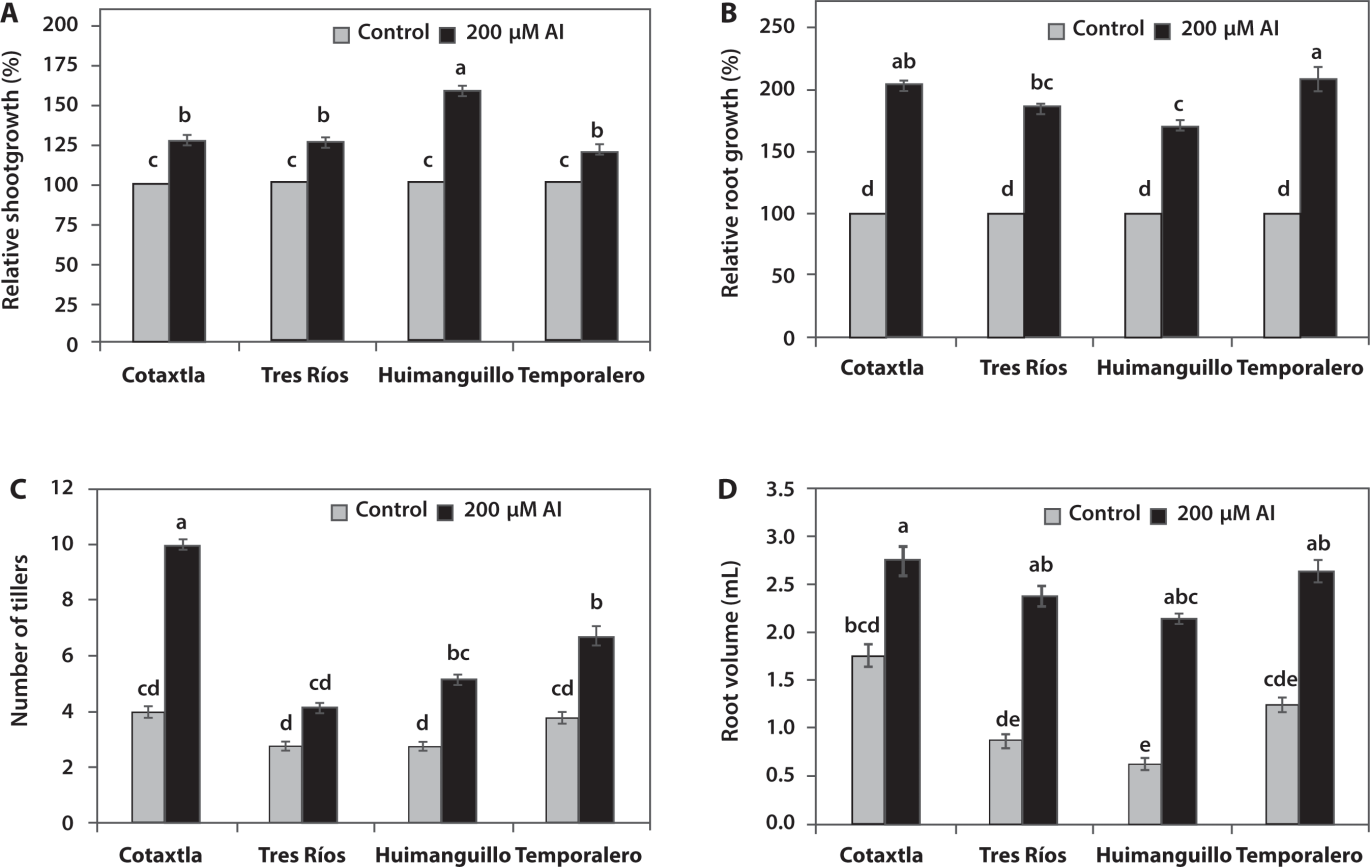
Stimulating effect of Al on rice plant growth. Relative growth of shoots (A; range: 100–159) and roots (B; range: 100–208), number of tillers (C; range: 2.8–10.0), and root volume (D: range: 0.63–2.75) of the Cotaxtla, Tres Ríos, Huimanguillo, and Temporalero rice cultivars under treatment with 0 (Control) and 200 μM aluminum (Al) for 20 days. The means of four plants ± standard error is shown. Different letters in each subfigure indicate significant differences (Tukey, *P* ≤ 0.05).

Al is one of the most abundant elements in the Earth’s crust, and its toxic form (Al^3+^) is solubilized in acid soils, affecting the most important crop plants. Many plants that thrive in acid soils have developed defense mechanisms that counteract root growth inhibition caused by Al. Moreover, at low concentrations, Al can stimulate defense mechanisms against herbivores, prevent Fe toxicity, and promote P absorption, thus increasing root growth and development in a hormetic manner [36,38, 59]. In this research, we confirmed that Al stimulated the growth of both the roots and the shoots in all four rice cultivars evaluated (Fig 1A). The relative growth of the shoots in plants grown with 200 μM Al was over 26% in the Cotaxtla and Tres Ríos cultivars, and 58% in the Huimanguillo cultivar, in all cases, in comparison to the control. The lowest shoot growth was observed in Temporalero with only 19%, compared to the control (Fig 1A). The most notable effect of Al was obtained in root growth. In Cotaxtla and Temporalero plants exposed to Al, root length was more than twice that of the control, while Tres Ríos exhibited 85% greater root growth and Huimanguillo 69% greater than the control (Fig 1B). Al also favored the development of tillers, mainly in Cotaxtla, where there were 2.5 more tillers than in the control. Huimanguillo and Temporalero increased tiller growth by 80%, while in Tres Ríos there were no significant differences with the control (Fig 1C). Like in the case of root length, Al stimulated root formation, increasing root volume, with increases over 100% in Tres Ríos, Huimanguillo, and Temporalero, with respect to the control (Fig 1D).

We have previously reported that Al increases P and K concentrations in roots, as well as chlorophylls and total soluble sugars in shoots [1]. Similar results have been reported in other plants like maize [29], *Quercus serrata* [37] and *Camellia sinensis* [60]. In *Quercus serrata*, there was also an increase in the concentration of soluble sugars, especially glucose, as well as abscisic acid (ABA). This suggests that growth stimulation by Al involves a complex signaling network where glucose has a key role as an energy source and as a signaling molecule together with ABA, and might be related with carbon (C) and nitrogen (N) metabolism to induce root growth in response to Al [37].

### Expression of Al-responsive related genes

In the Al-tolerant cultivar Nipponbare (*japonica* subspecies), the application of 500 μM AlCl_3_ stimulates ABA synthesis, while the Al-sensitive cultivar Modan (ssp. *indica*) showed no induction in the synthesis of this hormone during the first 24 and 48 h, but did so at 72 h of being exposed to Al [61]. These findings indicate some possible Al exclusion strategy mediated by the *STAR1* gene, with possible regulation by ABA in tolerant cultivars (i.e. Nipponbare). In the Al-sensitive cultivars (i.e. Modan), there might be a detoxification strategy mediated by *ASR1* independently of ABA and jasmonic acid (JA). *STAR1* encodes an ABC (ATP binding cassette) transporter of specific expression in the roots required for Al tolerance [15]. Nevertheless, this gene (*STAR1*) is also overexpressed in non-toxic concentrations ranging from 5 to 50 μM Al a mere 2 h after application, and its expression is induced specifically by Al [48].

**Fig 2:**
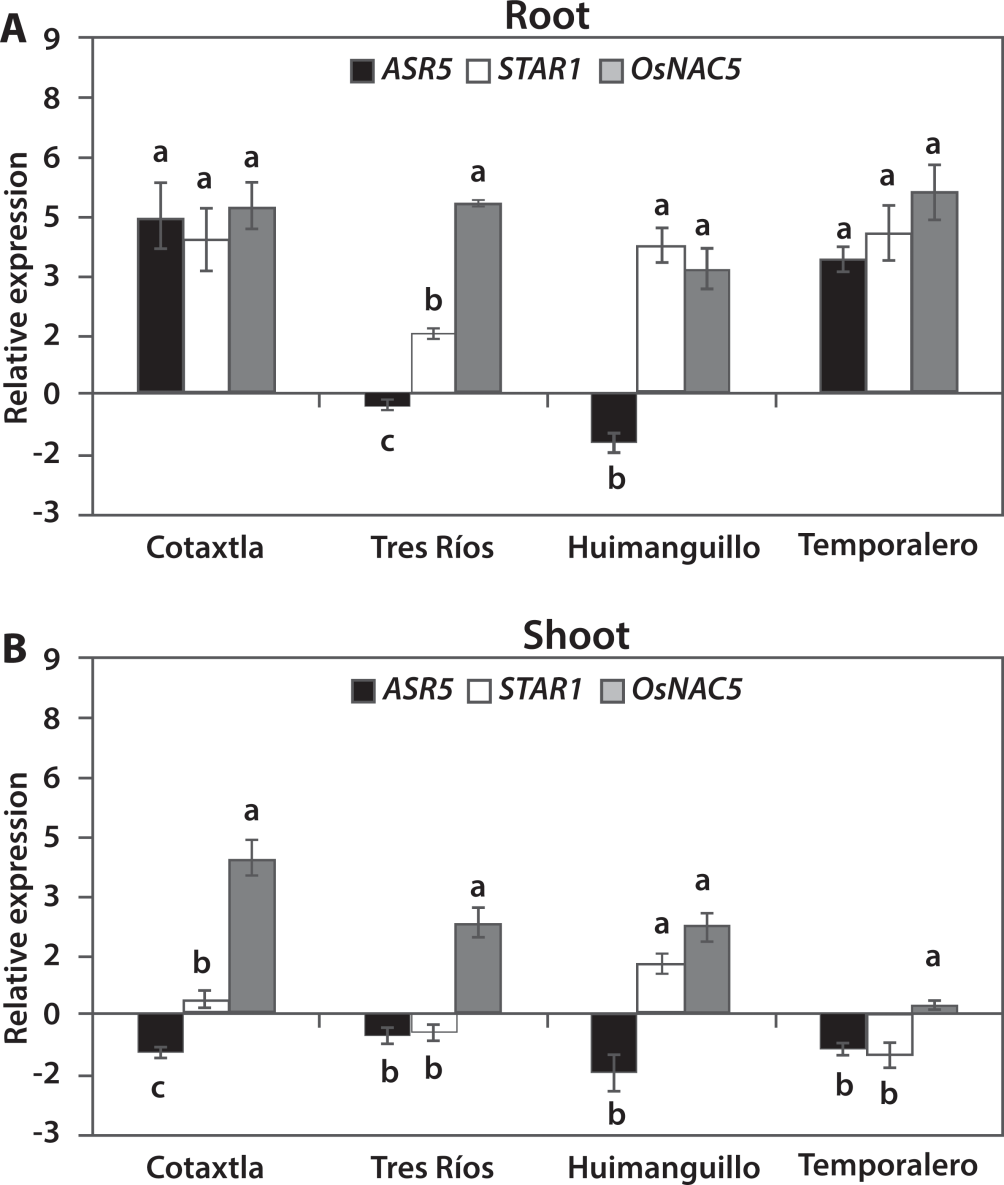
**Relative expression of Al-responsive genes *ASR5*, *STAR1*, and *OsNAC5* in roots (A) and shoots (B) of Cotaxtla, Tres Ríos, Huimanguillo, and Temporalero treated with 200 μM Al for 24 h.** Relative gene expression was quantified using the comparative methods CT (threshold cycle): 2^−ΔΔCT^, where ΔΔCT represents ΔCT_condition of interest_− ΔCT_control_. *Actin* (*Os03g50890*) was used as a reference gene for data normalization. The values are mean ± SE from three independent biological replicates. Different letters above the columns indicate significant differences among cultivars evaluated (Fisher LSD test; *P* ≤ 0.05).

In our study, the expression of some genes known to respond to Al was evaluated. In the roots, *STAR1* was found to be induced in all four cultivars evaluated: Cotaxtla, Tres Ríos, Huimanguillo, and Temporalero (Fig 2A). However, this gene showed no induction in the shoots of any of the four evaluated cultivars exposed to 200 μM Al for 24 h (Fig 2B). This agrees with previous reports and validates the experimental conditions of our study, since *STAR1* is expressed mainly in the roots and is specifically induced by Al exposure [48].

The *ASR5* (Abscisic Acid, Stress, and Ripening) gene is a transcription factor found in roots and shoots of the *japonica* Nipponbare subspecies, induced when plants are exposed to high Al concentrations (450 μM) [15]. We also found a differential expression of *ASR5* between cultivars of the *indica* subspecies, as this gene was induced in Cotaxtla and Temporalero roots, while it was not regulated in Tres Ríos, and was lightly repressed in Huimanguillo (Fig 2A). In shoots, it was found lightly repressed (<-2) in all the cultivars evaluated. Moreover, the exposure to 500 μM Al did not affect the expression of *ASR5*, either between genotypes (i.e. Nipponbare and Modan) or among exposure timeframes (0, 24, 48, or 72 h) [61].

A putative *NAC* gene from maize, identified with the accession number CA095885, which is similar to the rice *OsNAC5* gene, was induced in the roots of maize plants exposed to 283 μM Al, but not in those exposed to 75 μM Al [18]. In the presence of 200 μM Al, this gene was induced in the roots of all four cultivars evaluated (Fig 2A) and in the shoots of three cultivars, with the exception of Temporalero (Fig 2B). Under our experimental conditions, the Cotaxtla and Temporalero cultivars showed a similar expression profile in the roots, while Tres Ríos and Huimanguillo formed another group with similar expression profiles between them. With regard to shoots, Cotaxtla and Huimanguillo showed a similar expression profile, as did Tres Ríos and Temporalero. Both in roots and shoots, the expression profiles of Cotaxtla and Tres Ríos were different.

### Effect of Al on *NAC* gene expression in rice plants

**Fig 3:**
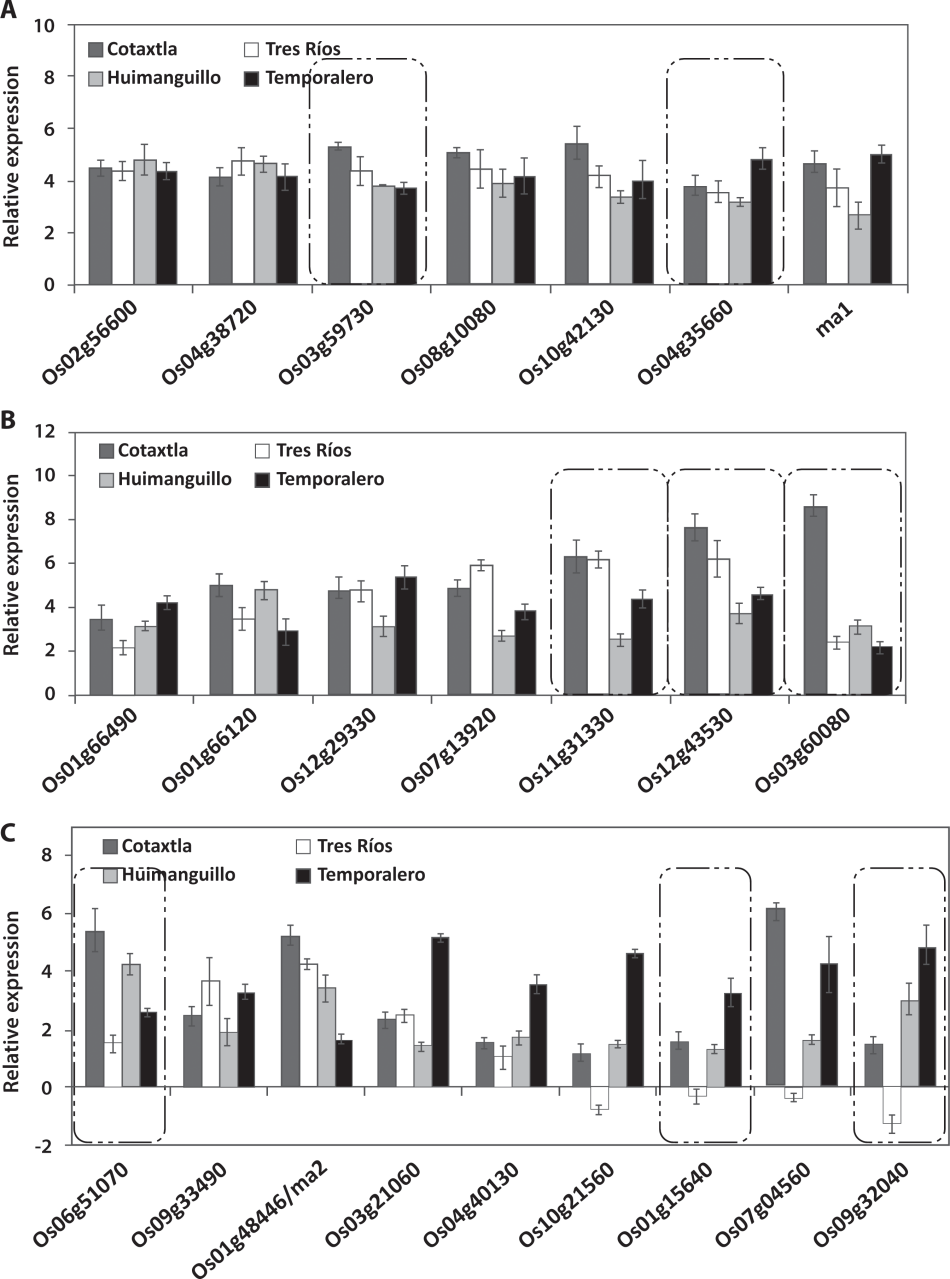
Expression level of *NAC* transcription factor in roots of rice plants treated with 200 μM Al for 24 h. *NAC* gene induced in roots of Cotaxtla, Tres Ríos, Huimanguillo, and Temporalero in response to Al treatment (A, B). Relative expression of *NAC* genes that were differentially regulated by Al (C) in all rice cultivars evaluated. Relative gene expression was quantified using the comparative methods CT (threshold cycle): 2^−ΔΔCT^, where ΔΔCT represents ΔCT_condition of interest_− ΔCT_control_. *Actin* (*Os03g50890*) was used as a reference gene for data normalization. The values are mean ± SE from three independent biological replicates. ma = missing annotation. Al-responsive genes identified as root-specific expression are highlighted in boxes with dotted lines.

In the present study we evaluated the expression of 57 *NAC* genes in response to 200 μM Al applied to the nutrient solution for 24 h. We found that 23 *NAC* genes were expressed in the roots, 14 of which were induced in all four rice cultivars (Cotaxtla, Tres Ríos, Huimanguillo, and Temporalero) (Fig 3A and 3B). The remaining nine genes were differentially regulated in the cultivars evaluated (Fig 3C). Of these nine genes, *Os10g21560*, *Os01g15640*, *Os07g04560*, and *Os09g32040* showed a very similar expression pattern, being repressed in Tres Ríos and induced in the other three cultivars. Moreover, eight of the 23 genes were identified to be specifically expressed in the roots of at least one of the four cultivars evaluated: *Os03g60080*, *Os01g15640*, *Os09g32040*, *Os12g43530*, *Os06g51070*, *Os11g31330*, *Os04g35660*, and *Os03g59730*, as shown in the insets in Fig 3. The remaining 15 genes were differentially expressed both in the roots and shoots (Table 1).

**Fig 4:**
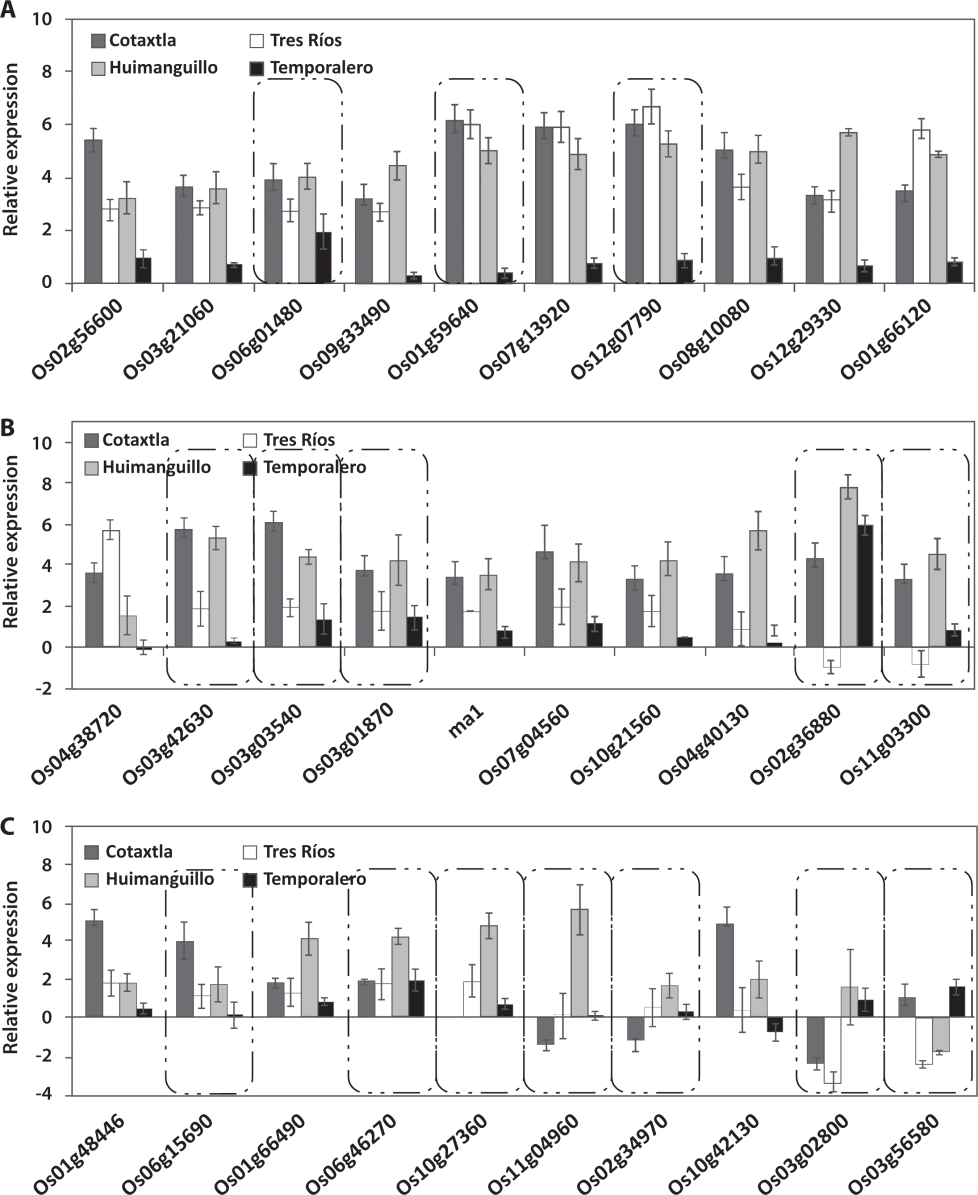
Relative expression of NAC transcription factor in shoots of rice plants treated with 200 μM Al for 24 h. *NAC* genes induced in shoots of Cotaxtla, Tres Ríos, Huimanguillo and Temporalero in response to Al treatment (A). Expression level of *NAC* genes that were differentially regulated by Al (B, C) in all rice cultivars evaluated. Relative expression of *NAC* genes that were differentially regulated by Al (C) in all rice cultivars evaluated. Relative gene expression was quantified using the comparative methods CT (threshold cycle): 2^−ΔΔCT^, where ΔΔCT represents ΔCT_condition of interest_− ΔCT_control_. *Actin* (*Os03g50890*) was used as a reference gene for data normalization. The values are mean ± SE from three independent biological replicates. ma = missing annotation. Al-responsive genes identified as shoots-specific expression are highlighted in boxes with dotted lines.

An analysis of the relative expression of the *NAC* genes in the shoots of rice plants was also carried out. We found 30 Al-regulated genes in at least one of the cultivars evaluated. Of these genes, 10 were induced in the Cotaxtla, Tres Ríos, and Huimanguillo cultivars (Fig 4A). Another 10 genes were induced in two of the four cultivars evaluated, with the exception of *Os02g36880*, which was induced in Cotaxtla, Huimanguillo, and Temporalero (Fig 4B). The rest of the genes were expressed in a single cultivar, with the exception of *Os03g02800*. Most of the genes were induced by Al. However, the *Os03g02800* gene was found to have been repressed in Cotaxtla and Tres Ríos, while *Os03g56580* was repressed in Tres Ríos.

All the genes regulated in the roots were induced in 95% of the cases in at least one of the cultivars evaluated. A similar behavior was found in the shoots, with the exception of the genes *Os03g02800* (in Cotaxtla and Tres Ríos) and *Os03g56580* (in Tres Ríos and Temporalero), which were specifically repressed in the shoots (Fig 4C).

The expression of the evaluated genes in the Temporalero cultivar had contrasting expression patterns, since 95% of the genes were induced in the roots (Fig 3); in this cultivar, a single gene was overexpressed in the shoot (*Os02g36880*), while the rest of them were not regulated by Al, under our experimental conditions (Fig 4).

### Multiple alignment of protein sequences: Comparison of NAC transcription factors between *Oryza sativa* ssp. *japonica* and *Oryza sativa* ssp. *indica*

The results of the previously explained *in vivo* analyses generated three groups of *NAC* genes as a function of their regulated expression in response to Al in the roots or shoots of rice plants (Table 1). Given that the expression of these genes was evaluated in *Oryza sativa* ssp. *indica* cultivars (Cotaxtla, Tres Ríos, Huimanguillo and Temporalero), being that the design of the oligonucleotides for the qRT-PCR analysis comes from *Oryza sativa* ssp. *japonica*, a base cross was done through a comparative bioinformatic analysis of multiple protein sequence alignments. This analysis was aimed at learning if there is at least one NAC protein from *Oryza sativa* ssp. *indica* that has an identity equal to or higher than 90% for each NAC protein from *Oryza sativa* ssp. *japonica*, whose codifying gene has been tested in our *in vivo* study (Table 1).

The results of the sequence alignments are conclusive (Table 2): each expressed protein of the *NAC* genes from *Oryza sativa* ssp. *japonica* tested in our *in vivo* study (Table 1) has at least one expressed protein of the *NAC* genes from *Oryza sativa* ssp. *indica* whose identity is equal to or higher than 94%, with the exception of *Os03g21060* (identity of 54%). Moreover, some NAC proteins of *Oryza sativa* ssp. *japonica* have a 100% sequence identity, E-value of 0, and 100% query coverage with respect to one of *Oryza sativa* ssp. *indica*. These sequences are: *Os06g51070* and *Os04g38720*.

**Table 2:** Best protein alignments between the NAC sequences of *Oryza sativa* ssp. *japonica* and *Oryza sativa* ssp. *indica*^a^.

ID *Oryza sativa* ssp. *japonica*	ID *Oryza sativa* ssp. *indica*	Coverage (%)	E-value	Identity (%)
*Os03g60080*	*BGIOSGA013814-PA*	99	0	100
*Os01g15640*	*BGIOSGA001951-PA*	100	0	99
*Os09g32040*	*BGIOSGA029518-PA*	100	0	99
*Os12g43530*	*BGIOSGA035784-PA*	100	0	97
*Os06g51070*	*BGIOSGA020507-PA*	100	0	100
*Os11g31330*	*BGIOSGA035327-PA*	100	0	97
*Os04g35660*	*BGIOSGA016453-PA*	80	1.00E-122	100
*Os03g59730*	*BGIOSGA009581-PA*	100	0	97
*Os03g56580*	*BGIOSGA013656-PA*	100	0	99
*Os06g46270*	*BGIOSGA023457-PA*	100	0	99
*Os03g02800*	*BGIOSGA011734-PA*	100	0	99
*Os03g01870*	*BGIOSGA011685-PA*	100	0	97
*Os06g01480*	*BGIOSGA022108-PA*	100	0	100
*Os02g34970*	*BGIOSGA008422-PA*	100	3.00E-155	100
*Os01g59640*	*OsIBCD003666*	100	0	99
*Os11g04960*	*BGIOSGA036980-PA*	39	1.00E-129	99
*Os06g15690*	*BGIOSGA022664-PA*	100	0	99
*Os12g07790*	*BGIOSGA036535-PA*	100	0	94
*Os10g27360*	*BGIOSGA032901-PA*	100	0	97
*Os03g42630*	*BGIOSGA013151-PA*	100	0	99
*Os02g36880*	*BGIOSGA008492-PA*	95	0	99
*Os11g03300*	*BGIOSGA034713-PA*	98	0	100
*Os02g56600*	*BGIOSGA009257-PA*	91	0	100
*Os03g21060*	*BGIOSGA026407-PA*	95	7.00E-119	54
*Os10g42130*	*BGIOSGA033482-PA*	99	0	99
*Os01g66490*	*BGIOSGA004951-PA*	100	0	99
*Os07g04560*	*BGIOSGA025120-PA*	100	0	99
*Os09g33490*	*BGIOSGA031062-PA*	100	4.00E-179	98
*Os07g13920*	*BGIOSGA024543-PA*	100	0	99
*Os10g21560*	*BGIOSGA032080-PA*	100	0	99
*Os04g40130*	*OsIBCD014700*	100	0	99
*Os08g10080*	*BGIOSGA027481-PA*	98	0	100
*Os12g29330*	*BGIOSGA037382-PA*	100	0	99
*Os04g38720*	*BGIOSGA016546-PA*	100	0	100
*Os01g66120*	*BGIOSGA000374-PA*	100	0	99
*Os01g48446*	*BIOSGA001029-PA*	100	0	99

^a^The protein sequences of *Oryza sativa* ssp. *japonica* were obtained from the PlantTFDB v4.0 database (http://planttfdb.cbi.pku.edu.cn/) [51], with the exception of *Os06g15690*, which was obtained from PlnTFDB v3.0 (http://plntfdb.bio.uni-potsdam.de/v3.0/) [52] and *Os04g40130* from the Rice Genome Annotation Project v7.0 (http://rice.plantbiology.msu.edu) [62]. The protein sequences of *Oryza sativa* ssp. *indica* obtained from PlantTFDB v4.0 (http://planttfdb.cbi.pku.edu.cn/) [51] have a *BGIOSGAxxxxxx-PA* identifier format, while those from PlnTFDB v3.0 (http://plntfdb.bio.uni-potsdam.de/v3.0/) [52] have an *IsIBCD0xxxxx* identifier format. The alignments were done using the BLAST v2.6.0 software (https://blast.ncbi.nlm.nih.gov/Blast.cgi) [53], using the default parameters.

### Analysis of *NAC* gene promoters in rice: Identification of *cis*-acting elements involved in Al responses

A pivotal component of gene expression governance is transcriptional regulation, which is controlled by transcription factors like those of the NAC family. The NAC transcription factors represent one of the most studied molecular constituents since they respond to several environmental cues, including Al [1,18]. The expression of these genes is regulated by *cis*-acting elements that are found in their promoter regions, which are mainly located 1000 bp upstream of the ATG start codon [31,63,64]. The interaction of the transcription factors with *cis*-acting elements allows the activation or repression of the transcription rate of target genes [65]. Therefore, the identification and functional characterization of these elements are important to reconstruct transcriptional regulatory networks [66]. To determine *cis*-acting elements in the promoters we analyzed the *Oryza sativa* ssp. *japonica* proteome, since it is the most studied genotype in relation to Al tolerance. When performing protein sequence alignment with the *Oryza sativa* ssp. *indica* proteome (Table 2), we confirmed that they are almost identical (>97% identity), so our results would not be skewed if we determined *cis*-acting elements of *NAC* gene promoters from *Oryza sativa* ssp. *japonica*.

In the *NAC* gene promoter regions that were identified in response to Al (Table 1), *cis*-acting elements were found in response to cold: LTR and C-repeat/DRE; heat: HSE; drought: MBS; anoxia: ARE; salicylic acid (SA): TCA-element; ABA: ABRE, CE, and fragment IIb; methyl jasmonic acid (MeJA): fragments CGTCA and TGACG; gibberellin (GA): P-box and fragments GARE and TATC; ethylene (ETH): EIRE; and auxin (AUX): TGA-box and TGA-element (Table 3). The *SNAC1* (similar to *Os08g10080*) [1] and *OsNAC6* (*Os01g66120*) [1] genes have been reported to respond to drought [45,21]. On the other hand, the *SNAC2* gene (*Os04g38720* and similar to *Os09g33490*, *Os12g29330*) [1] responds to more than one stimulus or stress factor: cold, drought, lack of oxygen, and ABA [21,67]. Importantly, these findings are consistent with our results, with the exception of those observed in *SNAC2* (Table 4). Actually, the response of plants depends on various factors and, according to the identified *cis*-acting elements, it is possible to establish that the response to cold by *SNAC2* is carried out indirectly by the action of phytohormones. Indeed, phytohormones play pivotal roles in promoting plant acclimatization to ever-changing environments by mediating growth, development, source/sink transitions, and nutrient allocation [68]. Interestingly, all Al-responsive genes studied contain at least one *cis*-acting element involved in phytohormones responses (Table 4).

**Table 3:** List of *cis*-acting elements found in the promoter regions of *NAC* genes regulated by aluminum in rice.

*Cis*-acting element^a^	Consensus sequence	Related stimulus or stress^b^
LTR C-repeat/DRE	CCGAAA	Cold
	TGGCCGAC	
HSE	AAAAAATTTC	Heat
MBS	CAACTG	Drought
ARE	TGGTTT	Anoxia
TCA-Element	GAGAAGAATA	SA
	CAGAAAAGGA	
	CCATCTTTTT	
	TCAGAAGAGG	
ABRE	CGTACGTGCA	ABA
	GACACGTGGC	
	CCGCGTAGGC	
	CGCACGTGTC	
	AGTACGTGGC	
	TACGTG	
	GCCGCGTGGC	
	ACGTGGC	
	CACGTG	
	CCTACGTGGC	
	GCCACGTACA	
CE1	TGCCACCGG	
CE3	GACGCGTGTC	
Iib-Fragment	CCGCCGCGCT	
CGTCA-Fragment TGACG-Fragment	CGTCA	MeJA (Methyl jasmonic acid)
	TGACG	
GARE-Fragment	TCTGTTG	GA (Gibberellin)
	AAACAGA	
P-box	GACCAAACTCGT	
	CCTTTTG	
TATC-Fragment	TATCCCA	
EIRE	ATTTCAAA	ETH (Ethylene)
TGA-box	TGACGTAA	AUX (Auxin)
	TGACGTGGC	
	AACGAC	
TGA-Element	AACGAC	

^a^Definitions of *cis*-acting elements: LTR, Low-Temperature Responsive element; DRE, Dehydration Responsive Element; HSE, Heat Shock Element; MBS, MYB Binding Site involved in drought-inducibility; ARE, Anoxia Responsive Element; ABRE, Abscisic acid Responsive Element; CE, Coupling Element; EIRE, Elicitor Responsive Element.

^b^Abbreviations of phytohormones: SA, Salicylic Acid; ABA, Abscisic Acid; MeJA, Methyl Jasmonic Acid; GA, Gibberellin; ETH, Ethylene; AUX, Auxin.

**Table 4:** Frequency of *cis*-acting elements found in the promoter regions of Al-responsive *NAC* genes from rice tested *in vivo*.

Locus ID	Total	Responsive to^a^										
		Cold	Heat	Drought	Anoxia	SA	ABA	MeJA	GA	ETH	AUX
In genes displaying root-specific expression												
*Os03g60080*	12	1		1	1	1	0	4	3	1	0	0
*Os01g15640*	7	0		0	0	1	0	1	4	0	0	1
*Os09g32040*	3	0		0	2	0	0	0	0	1	0	0
*Os12g43530*	7	0		1	1	1	0	1	2	1	0	0
*Os06g51070*	5	0		0	1	0	0	0	2	1	0	1
*Os11g31330*	7	0		1	1	0	1	0	4	0	0	0
*Os04g35660*	3	1		0	0	0	1	0	0	1	0	0
*Os03g59730*	12	0		0	2	2	0	1	4	3	0	0
In genes displaying shoot-specific expression												
*Os03g56580*	11	0		1	0	1	2	3	2	1	0	1
*Os06g46270*	4	0		0	2	1	0	0	0	1	0	0
*Os03g03540*	6	0		0	1	1	1	0	0	3	0	0
*Os03g02800*	11	1		0	3	0	0	0	4	2	0	1
*Os03g01870*	6	0		0	1	2	0	1	0	0	0	2
*Os06g01480*	4	0		0	1	0	0	1	2	0	0	0
*Os02g34970*	4	0		0	0	0	0	0	2	1	1	0
*Os01g59640*	8	0		1	0	1	0	2	4	0	0	0
*Os11g04960*	8	1		0	1	1	1	1	2	1	0	0
*Os06g15690*	7	0		2	1	1	0	3	0	0	0	0
*Os12g07790*	8	1		1	2	2	0	0	2	0	0	0
*Os10g27360*	13	0		0	1	1	0	4	6	0	0	1
*Os03g42630*	4	0		2	1	0	0	1	0	0	0	0
*Os02g36880*	10	0		0	0	2	3	1	4	0	0	0
*Os11g03300*	6	0		1	0	0	1	1	2	1	0	0
In genes expressed both in roots and shoots												
*Os02g56600*	6	1		0	0	0	1	2	2	0	0	0
*Os03g21060*	17	1		0	3	1	2	7	2	0	0	1
*Os10g42130*	3	0		0	0	0	3	0	0	0	0	0
*Os01g66490*	6	0		0	0	0	0	4	2	0	0	0
*Os07g04560*	13	0		1	1	2	1	1	4	2	1	0
*Os09g33490*	7	0		1	2	1	4	0	0	0	0	0
*Os07g13920*	18	1		0	4	3	0	0	8	1	1	0
*Os10g21560*	7	0		2	1	1	0	1	0	1	0	1
*Os04g40130*	7	1		0	1	0	1	1	2	1	0	0
*Os08g10080*	8	0		0	3	1	0	1	0	2	1	0
*Os12g29330*	5	0		0	0	1	1	0	2	1	0	0
*Os04g38720*	9	0		1	2	2	1	1	0	1	1	0
*Os01g66120*	20	1		0	2	1	0	8	6	1	0	1
*Os01g48446*	9	1		2	1	0	1	0	4	0	0	0

^a^Abbreviations: SA: Salicylic Acid; ABA: Abscisic Acid; MeJA: Methyl Jasmonic Acid; GA: Gibberellin; ETH: Ethylene; AUX: Auxin.

### Analysis of *NAC* gene promoters in rice: Detection of putative motifs

**Fig 5:**
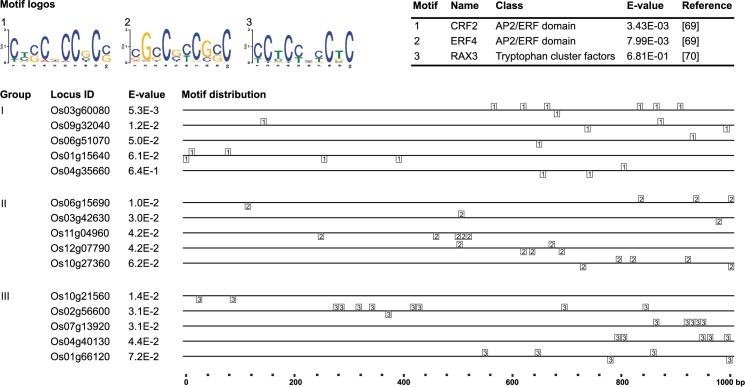
Analysis of putative motifs found in the promoter regions of Al-responsive *NAC* genes in our *in vivo* studies. The sequences were separated according to the differential expression of the genes, where Group I encompasses genes that were expressed exclusively in the roots,; Group II includes genes that were expressed exclusively in the shoots; and Group III comprises genes that were expressed in both tissues. The motifs, 1 to 3, of each group were identified with regard to their function (Table on the top-right corner of the figure) and distribution. The promoter analysis was done using the MEME v4.11.2 software (http://meme-suite.org/tools/dreme) [55]; the distribution in the sequences with the MAST v4.11.2 software (http://meme-suite.org/tools/mast) [56], while the identification of functions was done with the Tomtom v4.11.2 software (http://meme-suite.org/tools/tomtom) [57].

MEME v4.11.2 is one of the most widely used bioinformatic tools to recognize the putative motifs in a group of promoter sequences [55], so it was used in the present work. The result of the detection of putative motifs (Fig 5) is divided into three sequence groups, according to the differentiated expressions of the Al-responsive *NAC* genes tested in the present study (Table 1): expressed exclusively in roots (Group I); expressed exclusively in shoots (Group II); and expressed in both tissues (Group III).

The putative motifs (Fig 5) are consistent with the *cis*-acting elements found in this research (Table 4). Thanks to this study, putative motifs of ethylene response (GCC boxes; motif AP2/ERF) [69] were detected more precisely (with an E-value of 3.43E-03) in the Groups I and II of Al-responsive NAC genes (Fig 5). Another putative motif with SA, ABA and GA response (motif RAX3) [70], but with lower statistical significance (with an E-value of 6.81E-01) in the Group III was found (Fig 5). Ethylene, ABA, MeJA, and Aux are molecules that can act cooperatively to regulate plant growth and development. Importantly, we found scarce evidence of the molecular mechanisms involving NAC transcription factors in direct response to Al toxicity, since neither *cis*-acting elements nor putative motifs previously reported [15] were found in the promoter regions of *NAC* genes here analyzed. Indeed, the corresponding consensus sequence (_A/G_GCCCA_A/T_) present in the Al-responsive gene promoters like *ASR1* and *ASR2* in rice [17] was not identified in our promoter analysis. Regardless, the present findings suggest consistency in the response of the *NAC* gene promoters to phytohormones, which suggests that these molecules can act as intermediaries in growth induction promoted by low concentrations of Al (hormetic effect). In fact, ABA might be a key component in the metabolism of C and N, which activates a signal transduction network induced by Al stimulating root growth and development in *Quercus serrata* [37].

### Plant tissues, developmental stages and the presence of phytohormones differentially regulate the expression of *NAC*, *ASR5*, and *STAR1* genes

**Fig 6:**
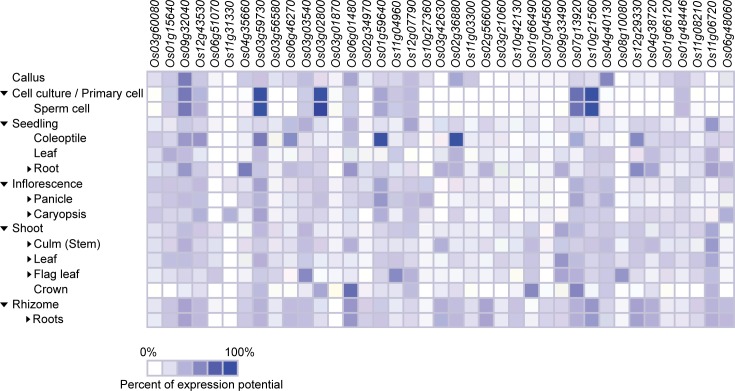
Tissue-specific expression patterns of *NAC*, *ASR5*, and *STAR1* genes in rice. All genes were selected based on their responsiveness to Al. The specific expression by tissue in cell culture, seedling, inflorescence, shoot, and rhizome was obtained from the Genevestigator (https://genevestigator.com/gv/index.jsp). Colors represent the intensity of the expression (percentage of expression potential), from white (0%) to dark blue (100%).

**Fig 7:**
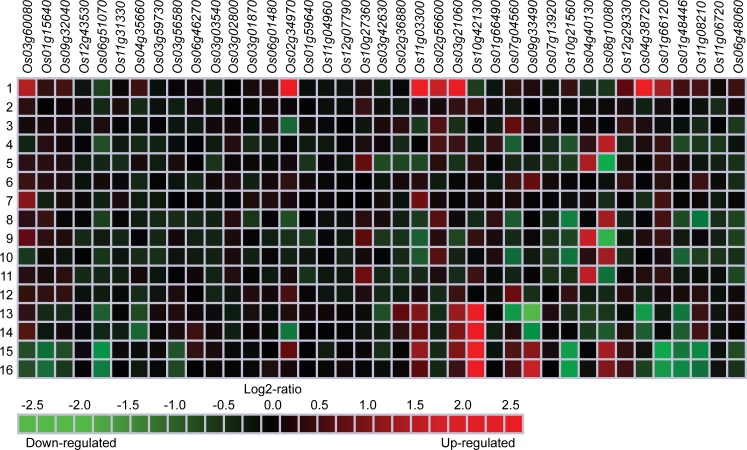
Expression patterns of *NAC*, *ASR5* and *STAR1* genes in rice from different development stages. 1: Germination; 2: Seedling; 3: Tillering; 4: Stem elongation; 5: Booting 6: Heading; 7: Flowering; 8: Milk; 9: Dough. Data were retrieved from Genevestigator (https://genevestigator.com/gv/index.jsp). Colors represent the intensity of the expression (percentage of expression potential), from white (0%) to dark blue (100%).

Genevestigator (https://genevestigator.com/gv/index.jsp) is a platform containing a great variety of precise and defined experiments that allow easily visualizing the expression profiles of genes subjected to diverse conditions. This tool was used in our study, to analyze the transcriptional expression of the *NAC* genes tested *in vivo* in the present research (with the exception of *Os06g15690* in Table 1), and three additional genes: *OsNAC5* (*Os11g08210*), *ASR5* (*Os11g06720*), and *STAR1* (*Os06g48060*) in different plant tissues (Fig 6) and development stages (Fig 7) of rice.

In general, all genes evaluated were expressed in all the tissues analyzed, although at different levels. The highest levels of expression of the *NAC* genes in rice were found to be in cell culture and seedlings. In cell culture, there was an induced expression of the *Os03g59730*, *Os03g02800*, and *Os10g21560* genes in sperm cells. In seedling, there was induced expression of the *Os01g59640* and *Os02g36880* genes in coleoptile (Fig 6). Regarding development stages, different levels of expression were observed in *NAC*, *ASR5*, and *STAR1* genes. The *Os03g02800* gene exhibited the highest degree of induction during the flowering stage (Fig 7).

**Fig 8:**
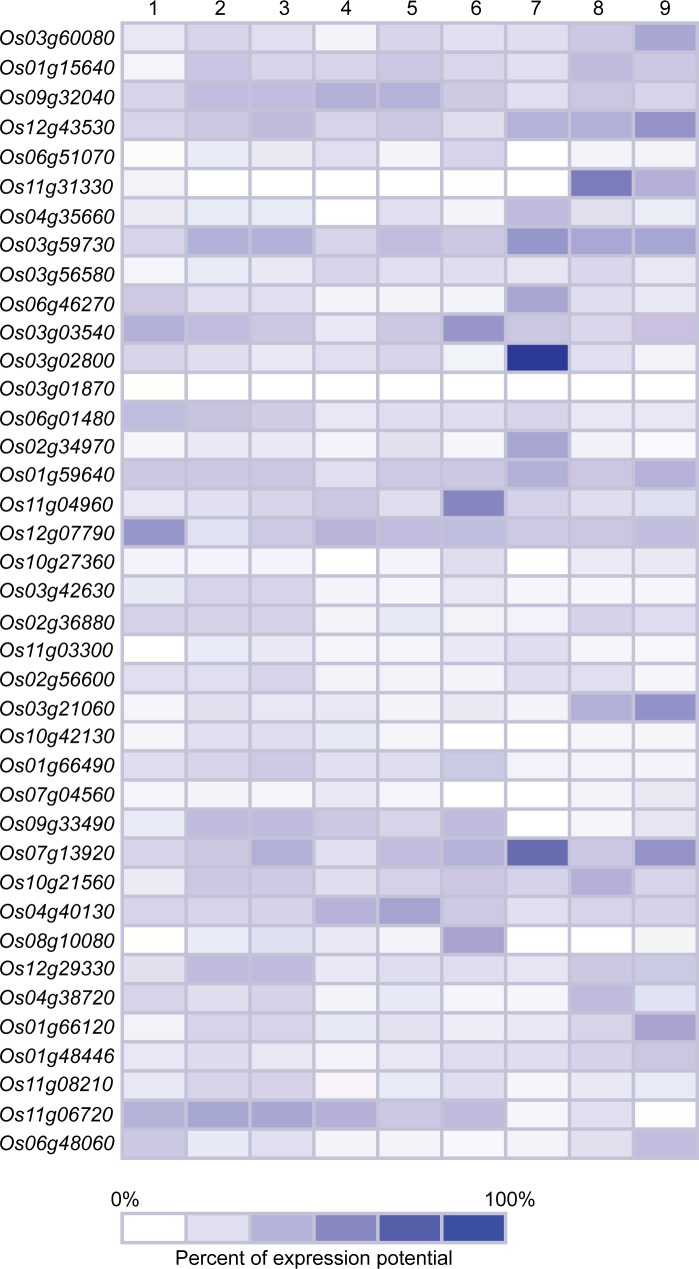
Differential expression patterns of *NAC*, *ASR5*, and *STAR1* genes in rice in response to phytohormones or phytohormone precursors. Expression data were retrieved from the Genevestigator (https://genevestigator.com/gv/index.jsp). Color saturation corresponds to the degree of up-regulation (red) and down-regulation (green) of gene expression in the specified conditions. Expression changes that were assumed to be of little significance were colored in black. Experiments 1–12: ABA (1); ACC (2); BAP (3); GA3 (4–5); IAA (6); JA (7); KT (8–9); NAA (10–11) and SA (12), were done in seedling tissues of *Oryza sativa* ssp. *indica*, while experiments 13–16 were done with trans-zeatin, where 13–14 were applied to the roots and 15–16 to the leaves of *Oryza sativa* ssp. *indica*. More information regarding these experiments is included in **S2 File**.

It has been reported that Al induces signaling pathways coordinated by phytohormones that regulate root growth and development in *Quercus serrata* [37]. Hence, our interest was focused on finding gene expression profiles data of *NAC* genes differentially regulated by phytohormonal variation conditions in rice. To do this, the data on gene expression deposited in the Genevestigator platform were used. From this analysis, it was found that all the *NAC* genes in rice that responded to Al under the experimental conditions (with the exception of *Os06g15690* in Table 1), plus three additional genes that have been proven to be Al-regulated, *OsNAC5* (*Os11g08210*), *ASR5* (*Os11g06720*), and *STAR1* (*Os06g48060*), are differentially regulated by ABA, aminocyclopropane-1-carboxylic acid (ACC; precursor of ethylene), 6-benzylaminopurine (BAP), gibberellic acid (GA3), indole-3-acetic acid (IAA), JA, kinetin (KT), 1-naphthalene acetic acid (NAA), SA, and trans-zeatin (Fig 8).

*NAC* genes have been proven to be Al-responsive [1,18]. Furthermore, Al can trigger signal transduction pathways upon which phytohormones act [37]. In tomato, the overexpression of the *Arabidopsis* NAC transcription factor JUNGBRUNNEN1 (AtJUB1) exerts conserved control over gibberellin and brassinosteroid metabolism and signaling genes controlling growth [71]. Importantly, *NAC* genes may be regulated by ABA-dependent or ABA-independent pathways because of the difference in their promoter elements [72]. In peach, expression of some *NAC* genes is induced by ABA and may regulate the first exponential growth phase and fruit ripening [73]. Using the Genevestigator platform, all the Al-responsive *NAC* genes in rice were proven to be activated by phytohormones in seedlings (Fig 8). When the rice plants were exposed to 100 μM ABA, the transcription of the genes *Os03g60080*, *Os02g34970*, *Os11g03300*, *Os02g56600*, *Os03g21060*, *Os04g38720*, and *Os01g66120* was significantly induced. Similarly, the genes *Os04g40130* and *Os08g10080* showed induced transcription when the plant was treated with 100 μM GA3. Another induction was detected in the transcription of the *Os03g60080* gene when the plant was treated with 100 μM JA. Also, with 100 μM kinetin, the level of *Os04g40130* and *Os08g10080* transcripts increased. These same genes were also induced with 100 μM NAA. Interestingly, when the plant was exposed to 5 μM trans-zeatin, the transcription of the *Os03g21060* and *Os10g42130* increased in both leaves and roots, which coincides with the present experimental data in presence of Al (Table 1). There is another relationship in the transcription of the *Os02g34970* gene in leaves. Furthermore, new genes with differential expression were observed: *Os07g04560* and *Os09g33490*, where the transcription rate was induced in leaves and decreased in roots. There were also changes in the expression of the *OsNAC5* gene in the presence of phytohormones, more towards repression than induction. With regard to the genes *STAR1* and *ASR5*, the changes in the expression profiles are not very significant, and tend more to repression in the presence of phytohormones. These findings support those previously reported [17] with respect to the fact that *ASR5* and *STAR1* directly intervene in the response to Al at toxic concentrations, apparently independently of phytohormones.

We have recently demonstrated that Al promotes plant growth and differentially regulates the expression of *NAC* transcription factors in rice [1]. Although the exact mode of action of Al in stimulating plant growth is still unknown, a few possible mechanisms have been proposed to explain it. For instance, Al may induce the synthesis of DNA in osteoblasts [74] and acts as mitogen in epithelial cells of mice [75]. In diploid cotton (*Gossypium arboreum* L.), *NAC* genes may regulate growth and cell wall deposition [76]. Moreover, Al promotes nutrient uptake by inducing the expression or activity of transport proteins (channels and transporters) and changes in the membrane potential and proton flux (H^+^) [77,78,79]. Indeed, Al can activate channels and Mg transporters in Al-resistant plants [80], and improves plant performance under nutrient deficiencies of B [81,82] and P [83]. Al also prevents Fe toxicity by reducing Fe content in leaves and roots [84,85,86]. In the case of stress responses, the protective capacity of Al against *Phytophthora infestans* is associated with the accumulation of H_2_O_2_ in the roots and the activation of the acquired systemic response depending on salicylic acid and nitric oxide [87]. In the aerial part of the plant, Al may increase photosynthesis and activate antioxidant defense mechanisms [88], as well as increase the integrity of the membrane and reduce lignification and ageing [89]. In addition, Al may stimulate the activity of the glutathione reductase and superoxide dismutase, at low levels of ROS [90], as well as that of nitrate reductase (NR) [91,92,93]. Likewise, growth promotion induced by Al has been associated with stimulation of NR activity, and increased glucose and ABA concentrations in roots [37]. Although ABA has been identified as a stress signaling molecule and growth inhibitor, this phytohormone is important for cotyledon, leaf, root, stem, and silique development and fertility [94], which may be associated with a concomitant increase in glucose concentration and high activity of NR, leading to cell proliferation and elongation in Al-treated plants [37]. In turn, most of these processes are controlled at the molecular level, and Al has been shown to regulate the expression of a number of genes related to growth and development, including not only *NAC* genes [1], but also others such as the malate transporter *AtALMT1*. Importantly, the *AtALMT1* gene may also be regulated by several phytohormones and hydrogen peroxide, suggesting a crosstalk among all these factors [79]. Summing up, NAC transcription factors play important roles in plant growth and development in mechanisms triggered by Al and phytohormones.

## Conclusions

The analysis of the Al-responsive *NAC* genes in our *in vivo* assays and their corresponding promoters demonstrated that these genes also respond to phytohormones; this, in turn, suggests that such organic substances might be intermediaries in cell growth and development induced by Al. Actually, ABA may mediate N and C metabolism during the signaling cascades promoting root growth driven by Al in *Quercus serrata* [37]. Indeed, according to our analyses of promoter regions of Al-induced *NAC* genes, phytohormones are involved in the hormetic response of rice in the presence of low concentrations of this element. Thanks to experimental data deposited in the Genevestigator platform (https://genevestigator.com/gv/index.jsp), we were able to gather crucial information to prove that the differential expression of the *NAC* genes in rice roots and shoots, in our *in vivo* assays in presence of Al, is also closely related with plant hormonal stimuli.

Our results confirm that the promoter regions of the *NAC* genes analyzed contain *cis*-acting elements that allow regulating their expression in the presence of a determined factor. In the case of Al, a signal transduction pathway is activated, with phytohormones playing a key role in the regulation of these transcription factors. These molecular interactions cause a differential transcriptional regulation among *NAC* genes, which evidently favors the growth and development of plants exposed to Al. In general, the expression of *NAC* genes is different between both tissues analyzed (i.e. roots and shoots) and among development stages of the plant (i.e. germination, seedling, tillering, stem elongation, booting, heading, flowering, milk and dough). Our findings provide a new insight into novel molecular interactions promoting growth and development in rice in response to Al. To the best of our knowledge, this study represents the first attempt to provide an in-depth evaluation correlating Al-driven responses mediated by phytohormones in rice, supported by *in vivo* and *in silico* data analyses. Nevertheless, further research is still needed to determine optimal concentrations of such phytohormones and Al that promote better plant performance. Importantly, Al has been proven to be a beneficial element to rice, while plant hormones play pivotal roles in promoting plant acclimatization to ever-changing environments. Therefore, interactions between the two factors could be of paramount importance in facing global challenges related to increasing food and energy needs, as well as climate change. The optimal combination of these signaling components could contribute to food security and sustainable agriculture in the near future. At the molecular level, we could confirm that some *NAC* genes are indeed Al-responsive. The corresponding NAC proteins represent key activators of diverse signaling processes, including aluminum and phytohormones, thus integrating multiple stress responses, which will be essential to breed broad-spectrum tolerant crops with high yields. It is expected that such crops, in turn, will be able to cope with environmental challenges in future climates.

## Supporting information

S1 Table Specific primers used for the qRT-PCR analysis of rice gene expression. (DOCX)

S1 File Promoter sequences of rice *NAC* Al-responsive genes used to identify motifs and *cis*-acting elements involved in Al responses. (DOCX)

S2 File Detailed description of experiments testing the effect of phytohormones on *NAC* gene expression. Data were retrieved from the Genevestigator platform available at https://genevestigator.com/gv/ [41].
(XLSX)
