# Supplementary material for: Expression patterns and promoter analyses of aluminum-responsive NAC genes suggest a possible growth regulation of rice mediated by aluminum, hormones and NAC transcription factors
Source: PLoS One. 2017 Oct 12;12(10):e0186084. doi: 10.1371/journal.pone.0186084 (PMC5638308; doi:10.1371/journal.pone.0186084)
Supplement: S1 File — (DOCX) [file pone.0186084.s002.docx]

**Expression patterns and promoter analyses of aluminum-responsive NAC genes suggest a possible growth regulation of rice mediated by aluminum, hormones and NAC transcription factors**

Hugo Fernando Escobar-Sepúlveda^1†^, Libia Iris Trejo-Téllez^2†^, Soledad García-Morales^3†^ and Fernando Carlos Gómez-Merino^1*^

**S1 File.** Promoter sequences of rice *NAC* Al-responsive genes used to identify motifs and *cis*-acting elements involved in Al responses.

***Promoters of genes exclusively expressed in roots***

>Gene_Response_In_Root-Os03g60080

Acccaacgagcctcacattcactgaatcatactagtcgtactactactactacaacactggaacggatagaaaacgataaatgacgccgcataatcgacaacgcatgggaccaaactcatgcttatctgctcttgtactcgtgcttatctcaaatccacctcattttacatgcccctttttgccacagcaactttcggtcccatcatggcatatttgcaaacgcaaaatagtttataaataaaattttttatatgcgtgtttttatagtgatataaaagcaacggttgaaagataaatttcgataaaaaaaccttaaaatcagctttaaatttaagattaaaaatttaaattttgactgataataagtattagcgaaaagatgatgtcctaatttccccttttcgctccaccccatgacgttgtccgacatctaaatactactcctaatcaacttgcaacctaagataatttagtatagtacacgtagtacagcctttttggagcagtaacaaaagacgcgcgggccacacgtccgtgcagttgcagccgccgctgcatccccaccgcgaaaaccacggcaaaatttagcggcggccgctgtggccgcctcacccccccgcgcctggctgtccacagcaacgcacgcacccgcacccgcaccgccgacccgaccgcggcgccgagctgtcccaattctgctgacctcggccgtgacgccatcctcgcacgggtccaatccccgacgcaggaggtggcctccttcctcgaaacccaccacctcaccaccacacgtgccccatttcatcccatctcctcttcttcccgcagccgccgactccgctttgactcatccccccgccgccgaaccttccagactacctccctctatatatccccccctccgcccgccatttctccccattcgagaaatccctcacaacccacaacattttcaaacaacgcaaagcagtagcagcagcgagaagcaagcaagaagcg

>Gene_Response_In_Root-Os01g15640

Cggggagccgtcgcgccgcgcggctctgattcttcccgttcccggaggtgagtgggttgactgactcctccccgctccgcgccgcgccgcgactggcctcgactctgcgtgtgtgtttggttaggtgatttggattgttccgcgcttgacgacgtccccgctcgttgttggggggtttcgtcttggtgcgggggatttttttgttgtttagcgttcgttcatgttaggggtagttgtttcgtctcgtctcgggaggtggggggttttagggtgattcttcgatcgagatcttcgtttaggggttgcgatcactcgccgattgcggatctttgccgcggaatttttcttattcttttttttttttttgcctcctggggttttcgattcggggggcagattcttgggggattcgagacgcgcgtaatggggattggttgcgaccccgtcgaaatccgagtggatcggtcggttggttggttgtgcgaagaaatggtgttcttgaagtagttcggtatttggggaattaggatagctgctcttccttcagtgcttcaataggatgataaatcagtgaattcccgattgttttttcttttcccattttagctagcctataataggatatcaccccacttttcaggtggtttaatgtgaaatttggagttattaccatattcctactaatcattttagaatttcctcccctgcttgctttgaacctgaaattgtcaataattagaaaggaggaagcatcattgttcatatggatttttgttagtttgcctccatgtttatcttctgtaaaatgcttggggatgttactccatgagcatactaaaatcgccaacgtttgtgttgagaatctttctagaaagatttataagatcgtcacttatgttctttagtttttaggcagtaagtcgttgtgctaaatacccatgccgttttagattctccctgatcatgtttgcttttcttgattcatgcacagatttgaacc

>Gene_Response_In_Root-Os09g32040

ggtgtttatttgacatcaaagtttactccatcaaacggaagactgaaacatctgttcctctcgctgtcaatgatagagcccgtatgtggaggcccctcaattcgtcggaggttacccttcactcaactttccaatgccaaccgctccccccggtgcacagagggatccaatcccatgatctatatgggacggacgcggtagtagctttggttccagaaagggtcatcattttgtacaatatttttcttcctcctggtagatggttggtcacattggtcaagatgatttgtgcttattagtcgtagtagagtgctggttgtcctactcctctgcatgctttttttttcccttcagtttcagttgtgtttgtgtcgtgtacaggcaggtgcgcgtgttgtgcaaatagcgctgctgttaaatctgtaatgctgttgctgaggcggcaacagatcggcatttgttgtacatgaacaacaatctccatatgtctatgggtgcataactgtttttgcctatggcccataattgctgcatattttcatctcatctcagataagtacgaatcattgtctcatgagttctaaaaggcttggaacatttgattcccatttacaaatgtcaaattgctgcagttctcgtatgcttcctagtactatctgcgttgcgttgactgacatgtgggcccgagggcgggtcgcgcgtggcgacgcgcgaagttaccgagaagacgcggaggagcgtaggcccacgcacatagcttttgttctctgtggaaggagctcgagggacaaagttagggggacataagtcactgacaaatggagcccaccctcatgggcccagatgtcagtgacacatgagactcctcctccgtgctccgtaagcttcgtgtttttttaagccggagggcgtcgagttgactcgccatttcgccatttcgtcgagaggttgctgtgccagtcaccaacccattccggcggcggcggcggaggagaccgag

>Gene_Response_In_Root-Os12g43530

gaaattattcttttatttgctaaaatgtactccatccattctaaaatataagacatagttaaacttgacatgatgtcttcaaaactactatttgactttcagtttctcacataatataatgttcgtagcaacaaattaaatcataactataaaaagctagtacattcaaatatcaatccaataacattgtttttatttatcaaataaattaaatttatatttgactaattgtttttcaaatattatgagagttgaatcttaaaatgcattgacatcttataagttgcactaagggagtaatttgggttgcatgtctcagttggaagaaatgagtagcacacctctaccaagtcaatcaatattatctgcctttgacttggtagaatgtcaatttcccatatatgtgcattgcctctgaataaactgagactctaattaaggtgttcaacggcctcccacaactctgaaaaccacgtgctacatatatattcaggcctaccactagtatatataacaatgtgataattttacttcgaaataaattaatccttcctgtatttaatttgctgtggcgagatcgaaataaagtatcgtcgtcactgaagaaaaatctcctgattcgacatataattgagttgagcaagtcatcagaggtacgacgatttatctgcagagacccatagggagagagagggttcaactcgattgacttagactcagcttgctcagacaaaaatttccagcatcttattattatcccaggctatagctttgtctagtataattaatcatcaaatgtgttctaatatggtcaaatatagcacattaatttattaatacagcagccatggccatacttctccatcaccatatgtaagcaactatgtatagctaacactaaacctcatacactatatataactagctgtatataggatcaaacaagttattatagctctgtccaattaagctagctagctagctactacaagattaagcc

>Gene_Response_In_Root-Os06g51070

cactagcaggaatgagctttaacgactgggtagtaatttaagcgtactttgccagatattttacttgtcatgtaaagtagtagtattactaaattaaaaagtgtgcttatcattttttatattaggagcatatctaattgctctaattgaattcgatcagtgtgattgatgctatatatagagaagtactactacaacagtaattaaatatctgcatttacaatttgttacctagttttttttattgatttgcttttatctagtactagctttttaaatcgcgcttgagggaaattgttccactgtatatttttgggcaaagtccattgagcaaaatgtagtggaattttaacagggaacacaagcacaagaaagacagaaaacctaaccctacaaatttccttttggtacgatttgcaacaggctttggacatcaacttgcattagacccagtccatcctctcttttcatcgttgttttagaaaaaaaggaaaacttgtcctcagctctagcactagcatgcccaagtcgatgaggtatgtgccttaattaactaacattcagtcagttagtcaccggatagctactactactaacatgcaatgtagtagtattaaattaatttagttaatcaggtagtgggcggcgcagattaaagcaagaaattccgatgtttgatgagctacgcagctaggacaggatgagacagtgaatggcagagtccaagtcacacacactacactagaatcgatgtgtcgtcgtcgtacgtttgtccagtaaaagtcagctctcgcatgactacgacgatggaatggaatggtgaccacttgctttgctgctacagtacttcctactgttacatatgtcatcgtcatcttctccaacgccggcctatatataccaagcaatttaacctctccttcctcccccaactcatcggtcgattaatctcgcctacaaacccatttacatccaatacagctagcatcaatacagagct

>Gene_Response_In_Root-Os11g31330

tatagtacctaaccttaaccaaatgaatgcttactatagggtgaaaagattagattggtacacaatatgtataatatgtctgtagccttttttacccaatacaaattttcaacaataactatatatgacaattagtgcaaggtaagaataataacaaaataaaatattactaatgaaaatggtaagaataaaaaaagagactaggacaaactgttgcaaattttctatcaatattacgaaatattattatcttaatacccaaaaaacattaaatagataaactttaatttagggacaacaatgcaaaaattagaaggaaaattcctttttttttcatgtctacccctccatgttaggattcttcacaacacaaaccttgtggtcccaccccaattaaactcatattctccaacacaagccccctatctttttactaggaaacaaaccccaaaaagctatctaacaatccaaataaggacacaaataaacatgcttgcaagcaacattcacacacctttaatttcagcctcaataagcatccacagttttcctagtcagcatggttgttcacatctggtcatagttatctctagcccccataaaagaccaaataggaagtgaggtagagtgaaattaaccggttgtatgagcttccaagaatggaaaagcacagaactgaacaaaacatggcattgggattctcatcccatcaacacaaacctcaatgctatctcaccagccatatcttggagatagagggagtgtgtgagggccagaaacttgcaaagggagaaagtgaggttgaatctccgtcacgtcactcacagttgcccatccatctgataaaggggtgtcccctgggctctataaatattgtccctctttgcctccatcaacatcactgcagtgcctttgagctttcgttctctagagtgtctagggtgaatcttgagcgagtgagcgagcgatctctgtgttgtgtagtttgtgagctagggtg

>Gene_Response_In_Root-Os04g35660

tatgcgtagcagcgtccaaacaaatctcaagagacagtttgctgcatgattgattgatattaacatggtatccaacacacatcaaataagagaaagaaagtcaaaactccagtctaatctccaccatagtttaatgacaggatgaggtttcacataacttgggcgtgcaacctaaacaagctggatgtgtgactatgtcatctcaccaattacatctcctgacttcagataagaaactctaatgactcgattatacgtttcattgacagttctcatgagcaaagagatggcactttataaattaatgtacctaatgtagcacaaaagcaatatgttatattatgatccatgcaagagctagtctgctatatattaaaaactttggatgatcgatgtctatgtaacctgatatatccaagctgagtcaatgaccaaatgcattctcaaaaggttaacctaatcaaggacaccgaaatgaacatcattgctacaaaggaactgtctttcttgtttcttcaactttgtcgataaaatataccaaacatgatgaggtgccatgcatgcatgccttgatgagcttaatttgttagttcccatcaatccttctcaatagttagtacagctgcatgcaatatattccctgaacatcgaggcgcagatactactgtctaaatccaacaccagcctttaattgcatagcatcatcagctccctcaatagaaaatgcagcatgggggaggaaaagaagaaagcatggcacttaagctgccaggcttctgaaggcaccatgcatgcctcagccgcctcaaaaacctttcaacgctccttgaattgcacgtctctctttctccacacattgcatacacgcatgcggccactataagtagcaccaaagtcaccacattaagcaagcacctgctctcatcttctccccgtagccttagcttccacccgcctgcctagtctatctacttcttctgccttacacttcacctcagcc

>Gene_Response_In_Root-Os03g59730

aaattgtttggactttgctgatcaagtgtgcacgtagaattcacgtcacttaaataaaattagcttgagctatttatgtctgctgactgaatggaatcgagaaaaaaaaagacattgtgtgggaaaccaaaacttgttaattagaaaatcagctatagactaaaccgaaggaatttagggtaacaacacgttaaaactggagatcaacttgtgatatggttcattcgataagatcgaccctacggatcatatcaaaccatgtatttttctttgggggaatctagtaaagaacctaactgttgttctttaattttaaaaatattagaatcgatgtattggtgagaataggtgtaggagtaaagattttgtgttgtacaatgtatttccaaccacttcacacgattcattggaaggatttggatttgcgaacagaacacttatacaaatcaccccatcatattgttacatccaatgtcgatttcgactactaaattctagttgaagcatcacaaaatattgaaatatgattccattatataaaaatgattcaactcaaaacccaatttcatcataagtatttcatgccatcttgtgctcacaaaaaaaaaaggcatcctagtctgaacatatattgtgtgccatttggcttcactcacctcaaatactttatggaacagaggactaatatagaagagagaggggcacatctttaatttgaagcatctgccatactatacattacctaaaaacagaagagaaaaaatgtggaagatagtaaagatcattagccgcaaaggtccaagcaaatgccacacaaatgaaccaatcacagtatttaatgttttcctgttatatatatagtgttggagacgtatcccaaaacactaatcataagtaataacagagttgtagagttgtaattacaatacaacttttttaactgtatcccaaaaagcccaaaaaaaaaaggtaggcgtcagagaaacggtgaatcattgca

***Promoters of genes exclusively expressed in shoots***

>Gene_Response_In_Shoot-Os03g56580

Aacaaaaggtatcttaaccaaaaaattcagaccaaaaacacatcgaattccacaataatttgtttttaggagtgtctatacacttcaggcgactgaatttttcaaaaatttccatcactttttctaccacttggatgatgaaaatccaatggttatcattactcaccaactacttattacttatcactctcaatgattgaacaataaaccgttctaacatacaaaaatttacaaaactacatgaaaaacattaacccataaaagtgtacaaagttacatgcaaaaaattcaaattatgtaaaattaaaataaaaccaacaacaaaattgcggttaaaatacgactaaaattataattttttagccgcctgaaaactagcaaatcccttgtctttttagggggaaaaaactcctttcccctgaacagtatctcagtcaaaagaagagatatcatcgatcggctctttgatttcttggcacccttacacccaacgacatggacgtggcatgatcaccaggtagtcaggcaccagcggatggccactgttgaggtgacatgtagcttaattagctagatgcaagtacaactttgagctgctttttctcgcagacaaattaaaaggcataaaaggatagcgtatagtaacaaactatagcttagctttgtacaaattaaaaggcatgatgttgctctgaagattcgtcaagacagcaagaggattcaaaccccatcgatcgatcagtgcatgacttggaccaagctctcatgcatttataaagccatagaagaatacccttttaattgctccatggactccttcacactcatatatatatagctagctttgctccttgcaaagaaaggccaatatctcaagagagctagctagagctccatttacaaccattacaagattgcatcagagaagatatcttcatagataattgttggagcaagaacacaagaaaacacgtaggttcaattataatcgatcgtggag

>Gene_Response_In_Shoot-Os06g46270

ttaatagaatattaccccatcgtccatccagttaataaataaataaaaagaatgtatttttagcgtttccagatgcatagtcagaaataaactattttttggacagagatatataaggattgtaagataagtcctcctatgtgttgtaggtccatgtttcatatatacagcttaaatatatatggttttttttctcttttgtacgcgtagcaattttagctttgtctgaactgaagcaactatcaaaaggcaattagtagacgactaactaactagtttctccctgcatatgtgaacacctagctagctacccttcgtgcccaacagcccataccttaccatgcatctgcatgcagcagcagacagcatcatatcgatgatccatccatctctggatcgatccagttgcaaatgcgaattaggcgaagcaataatcaaagctgagatatgatcgagccgagatagatcgagatcgatcatctgttaggtaatcactggttaggtcagtgtgtcctgaaattaatgcattgccactgaaagaaagaaaatgctcgacgaactgtgcatccatcgatccatggctgtacagtacatgcgcgcgtgcgtgtgcatcatcagcgatgcatgcgaacgagcatgacatgatcagccgctgagctcatcatcgctcgagatcacggcctaattagcgagttatggcgtgccacatggcatctggcacgcgaaccaagaaaagtctctctctcctctcctctcttcttcatcaacacacactggttgaccaaccagctagctgatcgagttcgttgtttaccatcctattaataccccttcctctctctcttctcccctcctgaatttctctcttggtagaggtagaattgatctcctttcttgttcttcttcgagttcttcttaattggagttcttggagcagcatcggtagtcgtcgtcttcgtcgatccagcatctcaaggaattcgaggaggagggggtgggc

>Gene_Response_In_Shoot-Os03g03540

cactgtctgttgaatgcttgtcgtctatgtttgttctaccgattcttcgtctcatctgttgcatctagtccgttctaatctcttcctatagctccttttattttcccttttgttcttctccttttttcatgtatagattatgcagttgggcagcagtgggttgtgatatactatgtgaattatcaagtgtgttgttattcgttaatttttcaagtagagtagtagataacccatatttattacataatacaaatggccatataagccacagagttgagggcgtttctgatcatgcacatgtaaagtttattgtttgaataatgagatcatgttgatataatagccaacctattgtactagttagttttggtattggctataaaatgacatggatgttattggagctagtagctggctacactattgaccttgctcttatatagcagaccatttttctgctgctgcaacagctataattcgttgctgactatagcccacatgcctctgagatatcaccagtcgacagtatcattcgcaggcctgtagctttcctacatttgtccctgctggcctcttgcccaggctctgccgtactttgcctttttatcctagaaagtccatagtgcaaacccaacatggctggattaaattttaccaaagcactgaaacccatacacactactgtagtagtatactccttgtcagtcagtctgtagctagtcattcagtatccagaccaaaccaatgctactagcccttctgtgcagatagcttttgcatttgtgtgtcctataaaaaatcgcaagatagccagcctgcttgcaccgaatgattcgagcacttgcatgtgttctttactactagtgccactcgcgagctatatcatttgatgcatgcatgcattgggtgtggatggatatttagaagacggtgttgctgaatctgatgcacggtgcatgcaattgcagtgcaggcacaggttggtgatcatatggacgcc

>Gene_Response_In_Shoot-Os03g02800

Gtatctccacaggtgaccagcttagtagcttactgtcggccagcaggctgcatccatcacatcggttaccgagcatggcatccactcttaaacacccagaacggaagaatgtacaggtaaacttgagctcagcattgtgcttctcagttgagagacagttaatcagctccttgacaaagtattagtactatagtacatggattagctgctaatcatgcagggcacacgcatcgaggcagggcccccggcaaaatctacatgggggcgcgtgtgtgaacgtgacgtaaaccgtcaaagggaagggggggaggctgccaggggcaaaaaagcgcgcgaatcgagtaccaaccttgggcccctcactctttccaacgagagctgactaggcacagccatggtggccccacaaagcaaccaatggcatctctccatctctggagctacagggcctcactttattggcagccaaagacaaggagagaagattgggatatttctctattcaccgctaaagctaactgacacaagacaacagaaagcaaaagcacccaaggtgagagagagagacatagagagcgagagagctgtacatggaggagtagtagcttcttgagaaagttggcaagaatccaaacatatacataaaatactctcggaaaacttgctataaagcacactccctcccgcagcccctttccctccctctcttgttcttcctctctcatgtgccattagccaatagcatcaaggtctcctctcttcctcctcgatcctcttcattttgtcttacttgcatgacattcttgttaactcatctatgtgttattgtatgttgcaggtttttgtacattgtagctgattataattcattcatatagctattcaaacaagttcggtggctgtccacattttagctttggatagccggcttgccagctgcctgcctagctctaagcatctcttgctgttggagttttgagaggaaggctagaggaagcat

>Gene_Response_In_Shoot-Os03g01870

aaatagatctttagtacaccaaccgggactaaatatagtaggtatcttttttcccggttggtggactaaaaataaagattaaaaagcagctctaaacttttgaaccgggactaaagataatctttaatcccggtttttattgcaaccgggactattgtggattttggctaaccgagcaaagatggtttatccaccagtaagatcacgacgggaagagattaatgtgcagtatagtcagttttgtgtcgttgaacagccttaagcgcccttaatgttgcaaatatacacgctcacgcacgaagtgattgataatagcacacattcaatgcaattatgctatagctaactattacgtacgtaccgatcgagcagcttgcatcgtatgactgtagagatgattcagactagaacagccaacgacatgtagtacagtacatctttgattaattagctcatcatgcatcgattgctatctacaagcttccaagatcaataagaatcttaattttccaatatatataaaaaaccagccagctgcgaataagataccgacaaagtatatactcccttcatttctaaatatttgacaccgttaactttttagcatatgtttgaccgttcatcttatttaaaaacttttatgaaatatgtaaaacttatatgaaatatacatataagtatatttaacaatgaatcaaataatataaaaagaattaataattacttaaaatttttaaataagatgaacggttaaacatattttaaaaagtcaacagcgttgaatttttagaaacggaggaagtatgaagttaggcacgcatgatggatatgcatgcgtacgtacgtagcttacctatagctaggtatatcttggtatgtgtatatatatacgaccatatccgagggcagagctgcacgtagtgttcgattaattcttgagctcatatattacttattcatccaaatagtattggtagcttgttgatcgaagaattaatcc

>Gene_Response_In_Shoot-Os06g01480

ctgcacatctctgaattagctaggatactaattactgtgataaacatggacgtggtagcacaatcataagttcccaagcacgcgcaaaatgtacatgtacatgtgtacataggatcgaagagagaaattaacaaataaggtgtttatatatacactcatcgatcaattaatcagcagatagctagatctcacccattcaaattaaattaagcacaaaagatcgagatggctggctatagctgcaacatgatatcatctctgtatgtaatcaataagagtacaattaatcgagcttaaaaatatgtacacgcaggcaagacatactatacacagatcgagatgtacacacatacagtacagtttgatctatctagctatacatgcagtagtagtagctagatagtgtgacaaattaaggttgcattggtagctagggacatacagcatgctgcatgcatgatgaggactttttcactcactatggtatgagctagtgagaggagagaggccggccggggaacattccacaaagagcaatgagtgagcatgcaccacatgcaggctgcaggcagcatatatatatattcattcattcattttgttagctagtaatgcattatgcgtgcgtcactccaaatcatcatcgatcgtgctcactctccctctcctgcttgctagctgttgcttcccttcgactgcatgcattcctatatatataccactactgcacttttcattcgatctatctaatccattatcacatgcattcatgcgtctccagccggccagccagccccagccaggtgttcagctggattaaaaaaaaaatcatgtatacagtaattaattaactgctctcgatcgatcgactacgtacgtacgtacatcactgtaagtattcgtagtttataacaatattgacatgcatgcatgactgtgatttcaggcaagcagaggcaggctctcaaatatatagatagatagcgatcgatcgatcgaa

>Gene_Response_In_Shoot-Os02g34970

atacaacaggaggacaacatctgggttgcttcttcttcaagtgatgcccttgtacttttgccacacaggtccagaaaaaatgtgcaacttgcttgctggcactcgtgtatataatggctggctgtggtcaaggtgcatatggcttgagaagggtgacgcctgcatgacatgagcaagaatgcaatggtcaggggggaaacaaatagtatacgcaatgatcatgaaaagaaatactgcactagagatgttgcctgtcacattaccttgttctgaggttttggggaatgtacattaaggagtgcctgtcacaatgtagtaatcagctcatgtgaaaaccttatcacttgataaaacttcaaacacttgatataataatctatacagtaagtaattaatacaggcttaaagaaattgctgatagaaaatccaattcaatgaggtaatgactaatcaacaaaaaagtactgatacctaccagctgagatctaatataagccaacaatccttaaggccatcaattatatcacaggctgctggatgaacacttttatcatgcatgtactatggaatgattcaattattatttctgcccccctctgaaaaactaatatatatctctgcacactactgcagccaacgggtacatagcagtgcatcatgaatccatgatccaacaaatgtctcctcatagtatcattatttccctccaaagaagaaaagagaaatatatagaatataagcatcgcaaaatgtcgccctcgcttggctgccatcctcctacggatcagcactcagcactccttgacttgcacgtctctctctctctctctctctctctctctctctctctacatctcactctttcacacaatcacacacatatgctccctataagtagcactcgctcaccaagatcagcaagcttcccatttcaaattcatcaatatcccagccaagttgcatttgactgcagtttgtaagttgcacttgtagttgtagc

>Gene_Response_In_Shoot-Os01g59640

ttttcttcttttttactcttatatatgtccatgatctctcaaatataagggtggtaatagacttaactttttagcctaaaaaaatttgagagcttaatttctatacacttttagcttaggaatatatttaaaggagtagataggattgtgaaaaaacccatagtccttgatccattacaaccgctaattaaatactaccaagattttacaccacagaatatgtggatctattgagacgcgtcgtcgctccgccttgaggatgggccgacgccgctttgccacaaagccgagccgtcgcccggggggggggggcgcctcgaggtgacgaacttgccactggtgatggagccgttatcaacctccgcgttagtgccgccgatgtcatcgctgttgtccgagagctggccagacaacaatgagagagctcactccgttgaaaatttgctgacaagtggggcctaccatatgcacgtcagcaaaatcagaggaatttttggtccatactaccataggacctactttgcacggcttttaatagttgaggggtagagatttatgtattgtggtttagagaccttagacaaacttgatgtaaagttaagggatgtcatgtgaacttaatattattttcaagtaaaattcacattgacatccggcccagcccaacacacatatggggcctggtcatgagctgattggctgacagatcatacgtactcccatcaaactcacaaataaagctccactcgtgcgtgcttgggttattcgagtcccacatatgccttgtagtattacgtaataataataatgtaattacagcagtcgtgataaatacggacactattcgttcgtgtttaaaaggttattttgaatgcccccggccccgccgcactatcatacgatctcgtgcgtgagatcgcttaaattgtaggcctccgactcatcagatcgcttaaatcgcggtaaattcgctgtccgaattgaatctccgtcgacggca

>Gene_Response_In_Shoot-Os11g04960

actagaagcaaccgaacacgcacagcacaaaacaaccagaccccaaaaaaaaatggaaacacaaaataggctagaatccatctacttcaccgcaaatcagcatcaaattacaatgtgaagagtaaccaatcgaaacgcaaggcagaatgagcactcgcacgcgcgaacacacacgcgcacacgagcacgagccaagcaatgcagtgccgggatacgagacgggggaggaggggatggaaatgaggcggagggcgttacatacacttgtcgatggactcgaagcactcctcctcctcctcgatcctctctgcatccatgagctggagctgcccgccttcgtcgtactgcttggacggcgccatctgctcacacagaccccccgcacctgcgtcaaaatgcgatcaggagacaacccaaccgaaaaatcaccccaaaaccctagcaaagacgtcgagcggaggccgctgcggcggagagaaccttcctcacctgtaggcggaggcggccggcgaccggcgacggcgaggtcggagcggcggcttgtaggagaaggaagcagcagaggcgagacgacaaacacactcgaagtggtgttgtgcttgtgccgttgcgttgcgttgcgtgcggcgaggtgggctacttgaggctctcgggccttctggaatcgtcttgttattgggcttctcctccgggaggaataattactactgggccgtttcacccagcccagcaggatagagaggcttgaggcccacgcggttcagaccaaaccaggcccaactaggccctccggactcaaaactcgctcatgggaacagaaccgaagcaaactaggattactaaacaacactattattccttatttccattacaactctctgtttcttcccgagaaagtttacagctctctctaattaactgcaattagttttagcatatataaatactaatttattatagcaatacccactttcctttttctgtccaattcgattcactt

>Gene_Response_In_Shoot-Os06g15690

Cagaaaaaaaattcaggcgggaaatacaaatgcaaatgcaaatcgaggaggcaaatccaacatggcggggagaggcatgatgccacgtaatacaaccatggatcacattaatggcgacgacgacgttggccggtgccaatttttccacaggggatttgctagatttcaggcacgtgccttttccccttccatatgcagcattaggacccgtttagatccctgccaaaatttttcaccctgtcacatcgaatgtttggatacattcggatacattcatgtagtattaaatataaacctaaaaataattaattatataaattgcgtgtaaattacgagacgaatcttttaagccgaattgctccatgatttgacaatatgatgctacagtaaatatttgctaatgacatattaattaggcttaataaattcgtctcgcagtttacagacaaaatctataatttattttgttattagtatgcgtttaatacttcaaatatatctctgtatatcacacgccaaaacttttggacctaaacacggccttagttgctggttggaccatcgggcctgggcttctataaagattgagctaacttttggggcttaatcgtctgatttggcccaacttggatatgggccgtttacaacatttggccccacacggattctcaagtcttacggggacggacacacctcatgcacttcatgccatctcgaccgtccaaaatgcaatcgaacggatgccgattcatctcaccatggacgcggtccagccaagcaatgccagcataaaccgcgtgcacctggtttatgtgctggtgtgtcctcgccgccgccatcgccggtcaactccggttcaaaaaaaaatttagagagaccaaccactcatcgtctctctacttccatccgcttcaaccttcaggtgcgccgcctcgccctcgtcggatccctagtagggaggggaggtctccgtctgccggcgggaggcgcgggcgccgcc

>Gene_Response_In_Shoot-Os12g07790

Ttagctttatctaacttcagaaacatgccaggaaaaaaggtttcggcttcaggaattcaactggccaccagcacgacaggatgaaaagaacatgtaccactactcaaaataacagctcaaaatgtaaatttataccaggcagattgtactaagatgagaaacattgcatcagacaagtgaggctcacagtatttatttccttcgaactgaaactatgcgggcagtgtatttggaagccctgcaaaatgctgtcgtctataaaccaggtatcctccattggcaaactcaagaccctgcaggtagcaaagatgataatccggaatccgaataaaaactgaattcgaaggcattttgttcaaaaccgaagattagaaaacgaacattttctgataaaaagaggaaatcgaactctagaagaagagagacccccaatctgaagggatttcgtcagtctgctccctgctgcgaatggccattccccattccccccacaaacgcggcgccgccgtgagacggtgctggccggatggagacagcgccgcccatgcgagcaagcggcggaggacaaagatagcggcagcgcactggcgtcgtgccgggctcgcctcaactgccgacggcggcgagccggcgacggcgtgggcgcagccgcagtcgcggaggaagccgccgccttcgcctcgccggcggctcatatatgggccgatttagctacaggccttaagaaattacagcccagaaagcccaaaaacgtaagcaaatatttccgactccgagtctttgaacattttttttatttttacagaaattttgaaaaaacaaaaaagttttttaaacattttaataaaaaaatttcctgagcccgcttagccgaacggcccgaacccaagtttgactcggtttggcaccatcaattttgccattaaaaggcaagaccaaaccggaaaaaggtccaaaaaaaaaaaagtcaaagcagtcaaaccaccgcgcggagaaggag

>Gene_Response_In_Shoot-Os10g27360

ccaccccctcatagcgctcacgtgttgttgcgccctccgtccccggtctattgacttcccgtggagcggtggcctctcgaagcccctcttcccgcttctgggaggtggtcttgggagagcacgtctgacgtgcagcgctcgctagcactgtcctctcggtcgccagcgatagccgggatgtgcacggaaaggacgacgcacaacgcattcttggtcttcctccacatcccgtccattgctcctagcacacgcatgcgtcgccactgccaccggtccccgggcctcttcctctgccgcctcgttccaccggccttcgcagcaatcgcttcgccctgcccagacaatgatgagaaaagtgatagagaggaaaaagggagagaagaggggaaagagagagggtgatgacgtgaacaccttgacacatggggtccatgtgggtcccacgctgacttagttgccacgtcagacaaaactgaggtcaaaactgcctaaggactcagagtgaactggttttgtaagttatggtatgacatatctagttttacggttggggatgtctatgtaatccgatgataagatgagggacctaatatatactttttccgaggaagtaaagctacggcccaaatggaataacggactgaactttgaacccaccggtttcttccaccgtacgcttcccccgttccgccagctggccagctcaacttgtcttggggttggggggggggggcaaaaaaaacaaaaaaaataaaaggaagcgaaaggaaaggagaatctcggcgcggcccccgccctcttaaatactacgccgccgcggccgcaaaagctccgagcacatagcacgcgcgcactcgtctcgccgcgtccacacccatcggctccaacaccgacgagtagctcggtcgccggggccgccgccgcgcgcgcgcgccaccgtgtcgcgacttcctccgtgaccgggccgatcgatcgcgaggcgcggcgcggcgaggccggc

>Gene_Response_In_Shoot-Os03g42630

tgtctgattgtgcattgtcttatccacttcggcagcagtggcagtataactaatgtgatgattagtactccctccctccgtttcaaaatgtttgacaccattaactttttagcacatatttgaccgttcgtcttattaaaaaaatttatgaaatatgtaaaactatatgtgtacatgaaagtatatttaacaatgaatcaaattatatgaaaaaaataaatgattacttaaatttttttttgaataagacgaatggtcaaacacgtactaaaaagtcaacggtgtcaaacattttgaaacggagggagtagtaagtagtataattatattgctgctgttagagaggcagctatagcttagtagctgctgttagagaggcagctatagcttagtagctctcgatggccatggccagtgccgggtcgccgttaatttcactgtgctggtggttgggaagtggttggagttggctggaattgaaacggtcgatcgatctcgacggcccccggccagcagctgacagctgcgttcctatgatcgaatcgatcgatcgaacgtgtggtctgatatgatcatggaagaagaatcgatcccagcaaataccttttcctgtcgtcgctgtcgcctgcgatattccttcccgtatcgtacgtcgctctcacctctctcacatatggccgtggttgttgcgaccctggaaattcatcagaacccccacgaggcaacgtaccttagctctagaaggagatcgatcagatgatgaggaggaagacgacgagcatgcgctgccactgatcgatcatcctatttgtttattgcgactgcctagcttgtcttcacgcacacatgtgcagtgtgcaaccggtatatataaaggtgtagcgggtgcctctgccatggacattagctagctcagctcagctcactgccactgccgctctgcctgccggcgatcgaggagaagaccggccggcggcgagctagcaggagagggctagtg

>Gene_Response_In_Shoot-Os02g36880

cgttgctacgtaagccaaattgatgtagcaaacaatgtttcgtcatattagtctaattgacgtgaccaaattattcaaatttaaaaaaaataagtgaagggtattttttttaaaaaaaaatggtgtgatgcggtgttgctccatgtggctacttgcattggtgcctgcgagtgcgagcatcactcattttgtggtatatcctgcggtatctgttcggttggcttggtgacctatgctaagttatttttcgaccagacactacgagtagggcagtacgtatatgagtagttttaccatcatttatatacgtatgcgtgcaaatgacatataaatcttttgcatattcttatctttttattattattaaacttatggctgtgtgtcttatgacagaagccggagatgtaatccatttctattatctatttttttttcatattcccaaaagtaaaaaatggactggaagatattaccacatacagggaactccttgcaaaaagggtaaccactttccaatattgattttgtctaataagtagttattgaactgagtgatgatgagctaaggtaaaagtagctagtccctgtaagtcaaaaaggttaatttagcggatcaaaccaaccccccttgcaagctgccggctggtgtgcacaatggctgtttgttccttgattgccacgtctctttccatcaaaaaaatcaatagtgcttccagtagtatttaagcccccaccatgaaccaacccctcagcttttgttttaatttcccccttaaccacttcatcacttctctcgtcgatctagtctaaaaggttagcaaatatcctctctctctctctctctctctctctctctctccttttctcgttcttctttgttcttgctagctagctcgctattgctaactcgtttgtgttaatattctgggtcctttgcttaatggttggtgttggttttttctgatgaattgctcgatcgtttatgtgtcaggtttagtag

>Gene_Response_In_Shoot-Os11g03300

Cgaatttcagcaaattttgaattgtatttctacttgtactctccttttctttttctccgattaatgtgggaatttctagtctccacaacgaacgtggtgactcctttcaaagctgttttaataatataataaatttactttgaatatatatatatatatataataaatttattttagaatgaagatagtagacgattgaaatatatatttgtgtggctctcatcttattaccttcatttgcatttacttatgaattctttgccactaattaaattttatataagagttagaattgactttttgaattatttgctactaactttaataacatcaagaattatgaagccatagagagtactatgaacaatatcacggttaacctacttttataatgtgatatcttctatatttttcttttttgaaaaaagataaatagtgaaactttgcaaataaatagtaatccgatgacaaaaaatgcaacgatcaaaggaggtagcaagtacaaacctatgtcaaactgaacttacgactaaatcgcttgcacgctgaaatttcaagttatttaatctcacatggttaaagtgtcaaatgtggtccagtctttggtactcacatcacgccacctactaataactagttaattgtctattgtgcccctagttagctctcgttaatcaattgcattgtagtaatccatccatgcacccaccacccacctctctttctcccgaacagcaagaagatgagccaagcccatggcgtctataaaagagagccctaccccctccatctcaaaaatttcaatccttcctcttccttagcttattagcttccttccttcactagtgccagttttcctcctacctaatctaagctagccaggtcgtcatcttcttccttcagctcacgctgaccaaacaccatctgttattctgtttgtttgtttttttaaaaaaaagaaaaaaaatctagctaggcgagccgattgaaggagctgagc

***Promoters of genes expressed both in roots and shoots***

>Gene_Response_In_Root_And_Shoot-Os02g56600

ttcatttacaaatatgccgttttacttgtaccacgaataaccgaaacgatggctccccgatcttgcacgcggttgaattgatatgcacacaaacacaatgataatgggcagctagagatcgatcgagctcgcgttttttccccaaatgcgtctctattgatagtgccatgtatgttgccgttgtcgctgcacttgggggatatgtacatatcgatcgtccatcatcaagtgggccggccgtggcgatgatctttcttgcgaccctggatctcctctctctctctctctctctgtccttcttcttccttgattcctcttcctccccaaccccacctgccctcctcctcctcttctctccagagagagagagagagaggtcgtcagtgaatgcgacgtcgtccgcgcacgcgtcctctcccgcgtcctccgcctcctcccgcgcgctgcgtacgtgacagcgccatctcctgcttgcagcagctagcaagcagcaggcgcaacagtattactgcagatagagctctcgaccgattgatcgattgctcactgccatatacatgatacttacataccgacgtactacacacagagatagagatcggagctatagctattgctattgctagccatagtatagtagtaccctttgctcctgctgctgctttgttgcttgcttccctacacttctctctctcagctgctagctgcgcatataattattccctcgccaagctatatatatttgcctcctgccttcttcccgagctcacacaatcgccaacctatacagatatatgtgctagctacttccaagttcctacctatcaatttctctcctcctcttcttcttctctgcatcaatctgcagcttaattcttagctatgtacatatagctagctaccacttccaaaatactccacagagatcaatcacatatagatatagctagattgctatagacagagagatcatcaaagaaatttagcaggtgatagccac

>Gene_Response_In_Root_And_Shoot-Os03g21060

Ttcaccacagcggtactatttgttggttcctggcatgaacccacaatgtgataagatacccgcaatcacagccatgtcctaagttaaaaacaactttgataaggtaattaattatagacgtgttgtaatcacgtcgtttttcaactagggatgcgctggtcatcttttcggtttgtgaccgtgctctattgtatcttagcaccttggtaccaattaagattttcttttcagcaactgtgttcctgttcactgtaaccaaacttcttttcaatctttaacaaatcttggccggtttctcggtgaaaaaaaaagaaaatatagacacgagttcgattccatggcgcacatattccgaggtgatatcctcttcggacgatgcaaaacggccgcatgtagggatccagtccgttggatatgccaactggcgaaattcatcccccacgcgaaaccccccaaatttaaccccaaaccgcctccacatacatcggccgccaccgcaccacgcggtccacgccaccaacacgtacacgtggcgcggccacggagttccaccggccgacgtcaggcgcgcctgacccgggggggcccacccccgcgcgcccaatctccggcgctatcacgccgggcgcgagggataacggaccaaacccaccccccgcgctccccatcctgcccccgcgaagcgccggaaaccacagaaaagccgcgcgggtggcatggcatcgcggccacgcatcatcaccctcaccaccattcaccgccaacgcaagcggacaaaaaatctgcgcgcataaaaggagcccctcccctcccgtccccaaaaattcgagccctcctccatctctcgcctcctcttgatacaagctcgaccctgatagatctcatagatcatagctgagaggggatcgagtggcttgatcttggaagtgagcagctatagctagataggttttgcttgtgcagggaggtgtgtgttgtgtgtgggtgactgccggagacg

>Gene_Response_In_Root_And_Shoot-Os10g42130

Ttccccccttccctgaatggtaagacagtatgattcttcagagaagaagaagaatatatcaccatgcattacatatgtcagtgttgccattatccaaaagcgatgtcattaatcatgcccagatatctgatcaatcagtggattaggcgctgccctaatcacagaggcaacctgacacgtcggtttggacaggagctaatcagagagaatggaagatgcagtagtaaagctcgagtagtgtgtgaatgtcttttgcggctacttggagttgcaactaccgtggtagagtagaggacgcttcgaattgcttcgctcgtacgaacatcatcgccgccttaccttagctttttctcctctcaacccctctatataaactcccctaccgtgtcaccttcccaactcccccaattcacaaaacgaaacccaaacactctcctattcctcctcttatttcttaattggtcagcacctagctagcaagaatctctcaactcttcttcttgttcttcttctcgtccataattgctcctccaattgatatctctgtacgtagttcagttcaagttcatcaaggagcctgaactgaattccttggttgtttagttcttgctagctaggggattgaccataggataatgatcttgaactccggaggcagcttcatcaacacctgtccgctggattccgagcagctcaagaccttctacttctggatcttcccaatcttcaatagatactgcctctgccgcctctaattgacaagcattcaaccatatatatgtgataaattgtgccaatctgcctcaagtattagcatttaggcaaattgacactacatctagctagctagctagctttgctccgattagtagtattgtttgcctcttctttccattgttaaccttcgattgaacaattaggtttgcgcttgcttgcccaagatatttgaataggttttgcaagtgtacatttgtctgaaattcagtcggttgatttgatc

>Gene_Response_In_Root_And_Shoot-Os01g66490

taagactaccgaagcaatctattagtccgcatacattagaaaagtctgaggtggagcagagatgaagcatgccaagagagaaggaagttgtcgactaaatagtcgacggtgatgcacgagcgcccaggctcatgcagccagtagtccgcttcatccttagacccaccaaaaatacagtgaaacaaagataaaaattagacccacatagatagataaagacaatacttttggagtaagtgccggagactattattaaaaacgttgtgtgcattacttgctatagttgagttggagatgtttagaaccggttgtgttattgcccttaccctaatataacgtgagatttgtagctagtaaattgaaacggagagagtaaaattgatagcacgcattaatgacgtagtggtatgctcatccatcaaacatcagacatcactacataagtagtagctcaaacactccccaagtccccaacccacaacgagagatagatagtagctaagccactgtacgtgcaagctagcggcacaacacaaccgcccatagctgtaccgccgcgcgcggcggagccgtccctttccctccacctatttaactcgcgcctccatcgctttccagtttccaccctcttctaccgctcacctcctagctctagcttgccccagctacgacgacgaggacgctgtccgtgtaccacatgtatgcgagctcgatcgacctatcgatctgagcgattccgatcggttatattagagagatcaagcatccgagctgctgctgtgttgtgggtacgtagatgtatgtatagtatagataccgcagccaccaccaccactaccacctctgctttttctaccctcgatatatattgtgctagatactagcctcttatccctttctctcttcttccgccgccgcgtcctcctccccaacttgaacactactactactcccccacggcgagctcgcgttctccgggatcaactagccctagcgacgg

>Gene_Response_In_Root_And_Shoot-Os07g04560

atataagaagctttaaagttgaacacagttattaagaaagtagatagaagtgaatggtggaatgttgtgattggatgagcagtgaaggtaagtggaaaaggtgaatggtggaggattatgattagttgggaaaagaatgttgatagagaaattatattttacgacggagggagtaagaatttaagaaagatatactgatcaatataatcaccattaagaagccggcagtcacattaaaccatgtagtgatattattaattaatcttctgcaagatgactcagtaaactttggcggcacgtctctcccgggacgtggctgaacgggcctgggagcatgtcttgtcatgtctaaatttcaaccgtaacaacaacatactgtagttaataaagccaacagacatgaataaattaaattaaaactctactcaacaacttttaccaaacacgcaccgtactaccaggcactgttaatctacgtcattgcatatattaatttttgccattcaattggccgtaccacgggcaaataatctatataaatgacgagatatgcatttatttcaaatgaaaagaattgtcaaaaaaaaattaggatatacacacacatgcatccaatgctgatatatttcgatatggacatggttgctagagagaagagaagtcaaagcaagtgacttggaccaagcaaccatgaaacttcatgactccatcctcttgcagctttcacaaaccaacagacagcttagtttagctcactatacacatccatcagagccatataaatatacatggctccatggccatctcctctcaacaacttcatcgatctcccctatatctttacagggatattaattccaagaacaacacaagaacaattttcgttctctagctcctagattgtttgagaagaggaggcgatcgatcgaatcgacgattcgatcgaacggccggcgaacactgatcatcaaagataaaattaatggtgccatctgcagag

>Gene_Response_In_Root_And_Shoot-Os09g33490

gggggcaggaagcaactcggcatattaagcaacggccaaaagagtgacatgattacaattagaagcaagaaaaggcgctaatgattcctcctaatccacacgaagtaccacggaagcacgcacaacacatggcgataatcagctctgccaattaagccagttgattagctcacaaggcaatcactgacaagaacatctgcacctgtgtgcgtgtgttcctcctgtcagtcacttgcaagctgcagtttgcatagggcacatcagggtcgaatccgatgtgcaaaaacgagcaaagtgccaatcctgaagtttgcttcatgatttttatcagaaaaaatgtttttaaaatgaataaccagacaaaaatgtccctaaaaacatggaatcatatagcaatcatagaaacaatagtactcaattttttttgtgcaatggccaatgggcatataaatccaagccatcgagtcaagtgaattgcttacatggcatgattaacaattgaccataaaccctctagtctatcttaaaaccaaaataaacaattaattaaatctctgtcaagcgctacttggattttttttctccactttctgaaaaaagagggaggagaagaaaaagtttttgtatcaatcagaatttaggagacatgtttagggtgaaacaagattggatcatgtcaaatcggagaaggaatcatactatttaagcaacaatggtggcaggattggagtctgctccacgccacatggatcctataaattccccgcccttttcttctccactccactcatcatcaaactctctctctctctctctctctctctgaatctctggatctctgaaaactctgaacattccaagaactcataagttacctgtgatctgtgaagcttgagctgaagctgtgacagtgtctcactgatcgttgatcactcagcttggctgctactgtgcattcactgcgtggttagctagctaactgagcagaagagctttagca

>Gene_Response_In_Root_And_Shoot-Al_Os01g48446

attttgatttacttttatcttacatttttattactattaaacttatggctgtgtgtcttatgacagaagccagagatataattcatttccattattaaaaaataaacttttatgagccagcgctaataatagaaaggagtcgtgccatataactcacctgattcacaagccaaaactagaaaaggaaatcaaaaagtgctcccaaccaattcagtcagatacatcgcgtacctttcacattataagatgttttgggtaaatagattcatatatatatatacacacacacttactttgtatatgtgtccaaattcgtatttatattagtgaatctagactaaagatgaaggaattttatagtatggaatggaggagccaaaacgtaatggaaaaaaaatacttttttccaatccaaggattcaaaagctctagaaaaaactcatggaaaagctaaattcatcgagattgctagtaataggccgtattgacgctactggcgcatacttttttttaagtccattagtaaatttgtcaattgggacctccagaaaatttggttggtagattccatatgctagaaaattaggaaaattctatactactggttattctacttctggagataccaatctcagagtacttctggaggcccagcacatcttacttctagaacggtcggcccagcccatcagagacaccaatcccggcacggactccgactccgactccaattcgcactccgaaactgacgccgcttcgtcgtctcccagccagcgcttgcgtaattagtacgtctcggctccgtttacgagccgagcccaactcggactcgggatttctacgcgtgaccgcgccccatataaacgcggccatcccccgcccggagtctttcaccattcttaccccgacgcggcgtctcctcccctctcctctccccttccctccatcgcatctcctcccgctccttcgatcccaaatcggccgccgccgccgctccgcc

>Gene_Response_In_Root_And_Shoot-Os07g13920

tagtccacgttgtgcagctatttgaatccatttacgaactcaagtctcaactgaataactgatccatctaactgatatctgatcttagaactccatttgcaacaatcgcatctgtgaggcgctttatccgtttatcggattagttgtactcagtagcagccttgatgtcttgatgcagttatcaagagaaacatcgtccaaaccactatcagcaaccttggtttcagataattgtcctgatacaagttttcggatgatggatagtaccgggtagaattcacaatattctgaagagtcttcttgccactaaaattaatctgcttgatatcttgagaagattccttttgacgcaaatatcttgagaagatcatagggaaacaataggaaatggaaacttcttctcattaaatacaacatctcgacatatgtctgaaactgaatgaaacacacgctcaaaatcaaaccattatactccaagagatgctatgttaaaatcaccaacaatattaaggtttttcccctatagtttgattgtcttcagaagtatactctgcttcccacctagaattatcctaaagatagaatccgtcagaaagtccaaagaatgcaggaagcatcttcagaatcaccatgagcagcaataagaatgcacagcctgaggcccctgatcgacagttcaaatttctgctaaaatttcaaaacaaatcaaaactaggttccaaaatcaggttgctaacacaagttatttcgttaagctttccaaaaacaaaataaaaatctacccgtgcggctgagcgaggcaacgcgcgcgcagcagggctgtctcatgctgacgtcaccccacgcatcccccctcctcctcttcgataaattcaaatcccgtccttgtgggggaagattcacccgcccgcttcctctcctctctctctctctctctctctctctctctctcgataccctaaaccctagaaattctcgagcgctcgcgatcgagactcca

>Gene_Response_In_Root_And_Shoot-Os10g21560

ggattcggatcgggggtgattttgagagggggggatcgggttcttgattcgaggtgcgattcgggaatcgggagttggagcgggggagggggaggaatatttcgtgtggggtttgtgcgcgattctccccgtcgcttttggggtaccatccaggggacgagggagtcgtttgatatggtcgcgttcatgcttctatttttgggtggcgatctcctgtaaaatttggacccggattcggggtggttctgtttgtggtcgagttttcctcgatttgattgctgagcggaatattttgttgggtgttcttgaatttttttttttgcgtcttatgtggttgattggtttgcgattcttcattagtttatcttcgccgcgtttgtgtaaatgtgggtaatgtaaagaacaaaaaaaaaattcagggactaaaattagtgtgttctgttatggcttcagcgttgctggtctgcagctgctgcggaagtagtaatagatgaggaaatgcccaccgatttttccagattgtttgatttcttcttgccctgttgttgtggccttgataagatccggttatggctacttattttattcttttatgccgtttaaatcaattagttcatgtatctttgctgtacaaattgtgtgttatcattttgagttgtggcattttgtcagtccaagattccctgagacaacgaaccttactaatgatggcggtgagcagctactgagctctgcgtttgctttacctgatagtgcttaatttggcaattgttagctctgcatagtgctactgtacacgtggtagtgttcagttttggtaggaagagcactaaatttaagtactgctatgtatcagttatttctttcagattgtttgaccacagaacagatacgttttcatgttatcaatgcacatccttttagaaatatgccttggtatttgtatgtcaactaaagaagcatgtcggctaaaattggttctattttttccaggacaatc

>Gene_Response_In_Root_And_Shoot-Os04g40130

gggaagtaagtgtgctttgctcgtaatactcctttctttttctgacttatattttgttacggttaagaacaaaatctgatttgcctttcctgaattgaatttcttgcaataggattttcaattcgggtcgcaggaaagcttcggctgggtggatggggatcacacgtaagtgatgctctgaggagttgtgggaagcagcggtgctgcaggaagacgccttcagggtttagggtctagggttcgccactctagtgagctgcaacactcttttagagctgcagcgctttttataagagtgacgaaatttggagtggtcaacccttcatataactgatctccgaaaattttgtatatggatttgcgtattaacaccagctctatgtagccgagcagtaagtatagtagtctctcttccccgtaaaaaaaaaaaaaagaaaaaaaatgtagcatctcaacctgaaaatgttctgtgagaacatgtttatgtgttgtaacctttgaaccttgtttatgtgttgtaacgtttgaaccatgtttatgcgttgttgtaacatgtaagccgctgcatgacattttccagtttattttcttctttctttctctctgtcttgttcaattttttctttctttttgaagtcttgttcagtttctgtacctctgcatgtgggaccgccttttggtagacgatctgctgagagaaaaatcaataccattgcccaactctgtagaaaactcacgaaaaaccctcctccgaagtccccaattccacatggcgccgcccacctccgccgccgccgccgtcgcggcggccgcccgcgcctcgccgacgtcggcggcggcgctcgcgctcttcaagtcggtgctgtccgcggacaaggcgctctcgccgctcgccgtgctcccgcacctcgacggcgcgccgtcctcgctgcccaacctcctgctcaccgcctccgccgccgtgcgcccccacgccacctccctccgcctctactcgcgg

>Gene_Response_In_Root_And_Shoot-Os08g10080

atctgttagagctgtaaaaagacctatttgcccctaactcattgtacatgtttttttttttgccgagggacatatgtattatatgaatacatatagagaaactgattgaatcatataacagcctatgattgctagcttcacgcgttggtagagacgtagtgaactagtgatctgttgactttgaaatgggacatatcggaaaaaaataaatgtacataatgaaaaaaaaaatcaaccccatacagttagatataaaacaagtttgaacatggtgcatggcactgagctcatatagatatagaaatgaataccaaaatattttactaaaacaggtgctagggtttattagtcatgggcattttagtcattaagaaaaaattcaatacaccaaacggatgcaccctaacggcttgtggacggcgatggcacgcagtagacgtttgggaaaactcgatggcatattattcaatttgacaaagtcagtggtatattgtagaagtggtagaaactcagtggcactgagtggattctctctattattaattaccatcatgtaaaatataaaaatggattgtgacatacgcacattccattctgacctgactgagctatagctactaattaacagctccagctcatgcaatgcaatgcaatgcaaccctgaaacaacaagcaatatatattgaaaaccatttagaccggtttatatattcagcaacagaatcgtactagtagcatataattagctcacatccaaatccttaatccgtaagcacactcccaaattaaacccggcaagttgcatattatccatgaaaaacccatcagttgctattgctatatactagcctctagctctagttgaccggcgagagtagtattaataccgctaccacgcgtagccgccgagctcgccattagcttgctattctctcttcttcttcttcttcttcttcttcttcttcttcttcttcttggtgtgattgaggtggaggaggag

>Gene_Response_In_Root_And_Shoot-Os12g29330

tttgtggggcccgcacatcctcagcccgatcccgtgcggcgcgcgatcccctccccccctccccaccgtgcgcgaatccatttccatcgtccgtgcgtaatcgtcgtccgttcgtcagcgaaaaactcgcccggtgaaaaactctcacgccacaccgttcaacaaaatccggctaggaaatatactccttcctcgtgagctcttcttgcccacgaaacactagtgctttgtatgtagtaatttttatacaacagataacttgattggtatgatctgtgaaatgatgttggtgtttggtgaacaaagttcatctaacaacacaaatagctacaaggatacaatttactactgatcacatacatagatatgtgtatattataattaattatttgattatgagatacagctagctaatggacaatctattgtactagtagttggctttagttatgggctattagccagtagcgggctatactattagccttgctcttatacttaacttatgccaaagagatacacaacaacttgcttgtacacactcccccaagttcaacccaccctttttgtttttctttctttccttttctttttcgtttcttcctcaccgtgccaaagatgaaaaaaatcactccaaatcgccgtagaaggcagcgccacaattaaaaccatccaaccatccatggtgccgtggtggtcgctgccttgcgccgcagagcaacgcgacagcgcaagtgacagtgacacagcgtcgtctcagctcagctcggctcccgagcttgagtcttcctcacgccactgctcccccctccccgccccgcgtcgaacaccttcctcctcccgctataaatccctctcgtctccctcctccacctctccccccgcctctactccccgcacacccgcgacaagctcagctcggctaggccaagacggtggcgagcggcggcgatctggtgctctgcttgggttgagttcttgattttgccgaggtgtatcg

>Gene_Response_In_Root_And_Shoot-Os04g38720

tctgatctgatggatcactagccactaaattaatccggtgattgatcatgtgtggaagcaagttttggctcgttacaaggtactattacataacactacgctgtcctgtatagctatctcatctatctcttcccatgcatttttcacctactagctagtactccatccgttttaaaatgtttgacaccgttgactttttagcacatgtttgaccgttcgtcttattcaaaaatttttgtgaaatatgtaaaactatatgtgtacatgaaagtatatttaacaataaatcgaatgatatgaaaagaataaataactacttaaattttttgaataagacgaatggttaaacacgtactaaaaagtcaacggtgtcaaacattttgaaatggagggagtagtaacctgtgatgagtttccttcatcacccacgtctctttctactaaaaagaccatcatgtagctcgcctctagtatttaaggggcaccaccacccctcccactttatttcctctatacaacgatttcctcttgtcaccctgaatctacttctgctgcaaaaggtaattagcatatcctaactatttatcacctcatccctcaagtgtgttcttctactttcttagatgtactttttaaacttttctgtgcataaacatctcatgtgctgattctttttgtgtgttctatgtgcttgcataccatgtccgtttggttcgattcgattttttaagcatccgtgtggtttgtttttgttctagcttagctagctagctagcaaagaggtgtcatggatacataccagctttaatttgatttggtttgctctacctttaaaagtgtgtgaatcatctccggtcctagtttatgcatatctgtgtgcatatgtacaaatgtttcaggacaggagttggcataacagctaacaaatcttacatatatagcagcaataagcaaggagcagttagccaggtaaagctctagctagctagcttaggcagca

>Gene_Response_In_Root_And_Shoot-Os01g66120

caacttaaacatgaaattaaattgtacatgtagcaaacaacctttataataacacaaaaaaaaccattttagttgaaagtggtattgctaaaagagttaaaagtatttcaatttagagcaacatgccagctacggctacagtcacaacccctttatcctttttctttcttccaaaatacaaacgcttaccttacagtgatggttctatgtcaaagctgtggggtccgttgaccccatagtttcttaccatcatcgtttagagtttcttaaattttctatatttatccgttgaaatttacacaaataatcccactaaaatatatagtaatgataatcgatgtcacagagtaaatttgctggctctgccactactcacctgtaaccccccaactatgccaccaaacacacataactcatcgcctcatcatcgtcatctatctccgcatgagaccgcatccttatcccaccacgtccccctcgcgctcacgcgcacagcaacaaagaaaaaaaaaaaaacccgtcccttttccctcgccgccccaccgctcccccaccccacgtgtcgccggcccatcggcggcggcgcctgcgtgggccgtgtggcccaccctgcggccccttcccgaaaacggaacgccccccccctcctcccctctccacgtcactgcgcggtgggcccgcgcgtgcgtccaagaagcgtgacgtaagcagtgacagaatccgcgccgcctctcggggcgcccacgtgtcgcggtcaaacggtcagcgcggggcgggggctcgcatcgcatctgctccacgtgtgcgctatcgcggctgcggccgcacgggccacacgtgtcgcttgcccccggctctataaatgcccggctcctcacccggaacaagtttcaagccctcctctcctcttcccaacactagtaggataaagccacagagagagcagtagtagtagcgagctcgccggagaacggacgatcaccggagaagggggagagag
